# Colorization Algorithm for γ-Photon Flow Field Images Based on the HSCN Model

**DOI:** 10.3390/e28090959

**Published:** 2026-08-27

**Authors:** Hui Xiao, Liying Hou, Jiantang Liu

**Affiliations:** 1 College of Automation Engineering, Nanjing University of Aeronautics and Astronautics, 29 Jiangjun Road, Nanjing 211106, China; liyinghou72@163.com (L.H.); liujiantang@nuaa.edu.cn (J.L.); 2 Shenzhen Research Institute, Nanjing University of Aeronautics and Astronautics, Shenzhen 518110, China

**Keywords:** photon imaging, parameter inversion, HSCN model, image colorization

## Abstract

γ-photon tomography provides a non-contact approach for reconstructing and visualizing flow-field parameters. However, the resulting grayscale images often exhibit blurred boundaries and weak texture features, causing conventional colorization methods such as DeOldify to produce cross-region color diffusion and boundary color overflow. To address this, this paper proposes a γ-photon flow-field image colorization algorithm based on the Hybrid Swin Colorization Network (HSCN). A hybrid dual-stream encoder composed of a Swin Transformer semantic stream and a central difference convolution (CDC) gradient branch is combined with cross-stage gradient injection and a spatially gated adaptive fusion mechanism to enhance the perception of high-frequency structures at flow-field boundaries and suppress color overflow. The effectiveness of the algorithm is evaluated in terms of colorization quality and flow-field temperature-parameter inversion using γ-photon flow-field images of two CFD-simulated flow patterns, a large-scale vortical wake and a horizontal wake. The proposed method achieves PSNR, SSIM, FID, and MAE values of 38.7422, 0.9372, 10.7344, and 0.0085, respectively. Compared with DeOldify, PSNR and SSIM are improved by 24.30% and 11.89%, while FID and MAE are reduced by 42.98% and 60.47%, respectively. In addition, HSCN achieved a MAPE of 12.65% across 15 boundary and temperature-transition locations in three representative samples, compared with 31.24% for DeOldify and 28.70% for DDColor.

## 1. Introduction

When a radionuclide undergoes β^+^ decay, it releases a positron. Within an extremely short time, approximately 10^−12^ s, the positron annihilates with a surrounding electron and produces a pair of 511 keV γ photons emitted in nearly opposite directions, approximately 180°. The propagation path of the photon pair constitutes a line of response (LoR), which contains key data such as the γ-photon time of flight, energy, and spatial position. A detector array arranged around the target region uses energy and time windows to select and record photon pairs that meet the specified conditions. The resulting data are referred to as coincidence events. An image reconstruction algorithm then statistically analyzes these coincidence events, reconstructs the distribution of positron annihilation locations, and maps the normalized annihilation probabilities to image pixel or voxel values, thereby enabling γ-photon tomography. When a radionuclide-labeled working medium is in motion, its flow field simultaneously generates LoR coincidence data. γ-photon tomography is used to establish the relationship between flow-field density and image pixels or voxels, and an image colorization algorithm restores the grayscale γ-photon tomogram as a color image containing flow-field parameter information. Furthermore, the strong penetration and electrical neutrality of high-energy γ photons allow detection to remain unaffected by high temperatures, high pressures, and electromagnetic fields. These characteristics indicate the potential of γ-photon tomography as a non-contact approach for flow-field-parameter inversion in aero-engine environments [1,2].

However, a γ-photon flow-field image is essentially a grayscale image with only a single grayscale-intensity dimension. It contains limited visual information and is difficult to use directly for condition monitoring in industrial settings. Deep-learning-based colorization methods combining CNNs and adversarial learning, such as DeOldify, have achieved good visual performance on natural images [3]. Attention-enhanced U-Net variants further improve feature selection and regional color consistency [4,5]. However, these methods are designed primarily for natural images and do not explicitly account for the probabilistic boundary characteristics of γ-photon tomographic images. Such methods generally assume that local pixels in natural images are strongly correlated and that color gradients are smooth, whereas the γ-photon imaging mechanism fundamentally conflicts with these assumptions. γ-photon tomographic reconstruction is a probabilistic statistical process. Each line of response is generated jointly by the pixels through which it passes according to the probability weights specified by the system matrix, and the coincidence counts follow Poisson statistics. After iterative reconstruction using methods such as ordered-subsets expectation maximization (OSEM), a positron annihilation location does not correspond to a single deterministic pixel in the image. Instead, it corresponds to a pixel region with a Gaussian-like distribution centered on the actual annihilation point. Consequently, a temperature boundary that is originally present in the physical flow field is smoothed into a continuously varying probability-density transition zone rather than a sharp physical boundary. Colorization networks such as DeOldify, whose receptive fields are formed through training on natural images, have difficulty modeling long-range temperature dependencies across regions. When faced with a broad, slowly varying probability-density transition, the network cannot accurately determine the spatial range over which colors should remain consistent on either side of the boundary. Colors in adjacent regions therefore encroach on one another within the transition zone and diffuse outward, producing boundary color overflow and compromising the structural integrity and visual continuity of the flow-field image. To solve this problem, this paper proposes an HSCN-based photon flow-field image colorization algorithm. A hybrid dual-stream encoder comprising a Swin Transformer and a CDC gradient branch jointly models global semantics and local boundary structures through cross-stage gradient injection and spatially gated adaptive fusion, thereby addressing boundary color overflow in γ-photon flow-field image colorization at the architectural level.

## 2. Relevant Theory

The core objective of image color restoration is to recover color information that is visually realistic and semantically plausible from images lacking color information. Its performance depends on accurate image-feature extraction and effective modeling of color-mapping relationships. Focusing on the core theory and key techniques involved in colorizing grayscale γ-photon flow-field images, this section systematically describes the basic principles of convolutional neural networks for image-feature extraction and the application mechanism of the Swin Transformer in image colorization, thereby providing theoretical support for the subsequent model design.

### 2.1. Hierarchical Feature Extraction Based on the Swin Transformer

Convolutional neural networks (CNNs) have long dominated image-feature extraction because of their local receptive fields and weight-sharing mechanism [6]. However, the inherent locality of CNNs limits their ability to effectively model long-range dependencies across image regions. This limitation is particularly significant for images with extensive spatial correlations, such as temperature and flow fields. To overcome this bottleneck, researchers introduced the self-attention mechanism from natural language processing into visual tasks and developed the Transformer architecture [7]. By calculating the similarity between any two positions in a feature sequence, self-attention enables information exchange across the entire sequence and fundamentally eliminates the locality constraint of convolution operations.

The Vision Transformer (ViT) proposed by Dosovitskiy et al. was the first successful application of a pure Transformer architecture to image classification. By dividing an image into fixed-size patches and treating them as a sequence input, ViT demonstrated the feasibility of Transformers in computer vision [8]. However, ViT uses global self-attention, whose computational complexity grows quadratically with image size, making it difficult to process high-resolution images. In addition, ViT outputs a single-scale feature map and lacks the hierarchical structure of CNNs, which limits its application to dense prediction tasks [9]. To address these problems, Liu et al. proposed the Swin Transformer. Its window-based self-attention mechanism and hierarchical feature-extraction design allow a Transformer to efficiently process high-resolution images and output multiscale features. The central innovation of the Swin Transformer is to restrict self-attention computations to local windows and enable information exchange between adjacent windows through a shifted-window mechanism. This design retains global modeling capability while reducing computational complexity from quadratic to linear with respect to image size [10]. Structurally, the Swin Transformer adopts a CNN-like hierarchy. Patch Merging modules progressively downsample feature maps between stages, ultimately producing four feature maps at different resolutions, namely 1/4, 1/8, 1/16, and 1/32 of the input resolution, with the number of channels doubled accordingly. These hierarchical outputs can be seamlessly connected to decoder architectures such as U-Net that require multiscale features [11]. Specifically, each stage of the Swin Transformer consists of a sequence of Swin Transformer blocks. Each block contains two alternating submodules: window-based multi-head self-attention (W-MSA) and shifted-window multi-head self-attention (SW-MSA). W-MSA divides a feature map into non-overlapping local windows and independently performs self-attention within each window. SW-MSA cyclically shifts the window partition boundaries, enabling features that originally belonged to different windows to interact within newly formed windows and thereby allowing information to propagate across windows. This alternating design maintains linear computational complexity while providing the model with the ability to establish a global receptive field [12].

Owing to its hierarchical multiscale representation and window-based attention mechanism, the Swin Transformer is well suited as the encoder backbone of a γ-photon flow-field image colorization network. It can model long-range spatial correlations among regions with different temperatures while providing multiscale semantic features for subsequent decoding.

### 2.2. Image Colorization Based on Conditional Generative Adversarial Networks

Image colorization aims to recover natural and realistic color information from a grayscale image. It is essentially an ill-posed mapping from a single channel to multiple channels because the same grayscale input can correspond to multiple plausible colorization results. Early colorization methods mostly relied on manual priors or reference images, offered limited automation, and had difficulty adapting to complex scenes [13]. With the development of deep learning, CNN-based colorization methods gradually became mainstream. However, when a network is trained using only pixel-level losses such as L1 or L2, it tends to predict the statistical mean of all possible colorizations, resulting in insufficient color saturation and blurred texture details [14]. Zhang et al. formulated colorization as a classification problem and alleviated color averaging by predicting a probability distribution over colors, thereby improving color vividness to some extent [15]. Nevertheless, pixel-level or classification losses alone still have difficulty producing realistic colors, and distortions are particularly likely to occur at boundaries and in high-frequency texture regions.

Generative adversarial networks (GANs) provide an effective means of alleviating these problems. A GAN comprises a generator and a discriminator that compete during training. The generator synthesizes images that are as realistic as possible, while the discriminator distinguishes generated images from real images [16]. Mechanistically, the discriminator acts as a learnable loss function that penalizes the excessively smooth results favored by pixel-level losses and encourages the generator to restore sharper boundaries and more realistic textures [17]. For image-translation tasks such as colorization, the original GAN alone cannot effectively constrain the generated result because its output is not controlled by the input condition. Conditional generative adversarial networks (cGANs) therefore introduce conditional information into both the generator and the discriminator so that generation is directed and constrained by the input image [18]. Pix2Pix, proposed by Isola et al., is a representative application of cGANs to image translation. It uses a grayscale image as the conditional input and jointly optimizes the generator with adversarial and L1 losses, demonstrating that a cGAN can significantly improve the visual realism of generated images while preserving structural consistency [19]. On this basis, ChromaGAN, proposed by Vitoria et al., further incorporates semantic-category information into the colorization process so that the color distribution of the result is more consistent with the semantic content of the image [20].

Although cGANs have achieved good results in colorization tasks, conventional GAN training is often unstable and susceptible to oscillation and mode collapse [21]. To strengthen the discriminator’s constraints on local details and high-frequency textures, image-translation tasks commonly use patch-based discrimination. Instead of producing a single real-or-fake judgment for the entire image, a fully convolutional architecture outputs a response map of local patch-level judgments. This encourages the discriminator to focus on details such as edges, textures, and local color distributions. The approach is consistent with the use of local patch statistics to constrain texture generation in Markovian GANs, in which discriminative constraints over local regions improve the realism of generated details [22]. In addition, adversarial training is often affected by instability, an overly strong discriminator, or large gradient fluctuations. Spectral normalization constrains the spectral norm of a network’s weight matrices and thereby limits the Lipschitz constant of the discriminator, suppresses abrupt changes in its outputs and gradients, and improves adversarial training stability [23]. Spectral normalization can be directly integrated into convolutional layers with limited additional computational overhead. These mechanisms provide the theoretical basis for the adversarial framework subsequently adopted in HSCN.

## 3. Colorization Algorithm for γ-Photon Flow-Field Images Based on HSCN

### 3.1. HSCN Model

Because of the probabilistic statistical principle of γ-photon flow-field imaging, the grayscale-intensity distribution in an image essentially reflects the spatial probability density of photon annihilation events rather than deterministic physical boundaries. Consequently, temperature-gradient boundaries that are originally distinct in the flow field are smoothed into continuous probability-density transition zones in the reconstructed image. When existing mainstream colorization algorithms process such images, a fundamental structural mismatch arises between the local-pixel-correlation assumption learned from natural images and the slowly varying probability gradients of γ-photon images. The limited receptive field of a CNN encoder cannot model long-range dependencies in temperature distributions across regions, causing the decoder to produce color overflow during color mapping. Specifically, color diffusion and structural blurring occur in flow-field boundary regions that should transition smoothly, compromising the visual continuity and structural integrity of the image. To solve this problem, this paper proposes a γ-photon flow-field image colorization network based on a hierarchical Swin Transformer and a gradient-aware dual-stream encoder.

The overall HSCN framework is built on the theoretical foundation of a cGAN. Through the dynamic competition between a generator and a discriminator, the framework achieves high-quality mapping from grayscale γ-photon flow-field images to color images. In this adversarial process, the generator receives an original grayscale photon flow-field image as input and attempts to render a three-channel color image with realistic colors and rich details. In contrast, the discriminator must accurately determine whether an input is a real image from the dataset or a synthetic image produced by the generator. The two networks compete and co-evolve during this process. The generator continually improves its ability to fool the discriminator, while the discriminator continually improves its ability to distinguish synthetic images from real ones. This adversarial process is the fundamental driver of model-performance improvement because it forces the generator to learn and reproduce the highly complex and subtle color distributions and texture details of real photon flow-field images.

To fundamentally solve color overflow caused by the limited receptive field of a CNN encoder, HSCN introduces a central modification to the generator encoder. A hierarchical hybrid dual-stream encoder based on the Swin Transformer replaces the conventional ResNet101 backbone. The encoder contains two parallel branches. The semantic stream uses the Swin Transformer as its backbone and exploits hierarchical window-based self-attention to capture global contextual information from the flow-field image, addressing the inability of a CNN encoder with a limited receptive field to cover correlations among distant temperature regions. The gradient branch is constructed using CDC. CDC adds a pixel-difference term to standard convolution, making the network more sensitive to local grayscale variations and enabling it to better preserve high-frequency structural information at flow-field boundaries. Features from the two branches are dynamically integrated through a spatially gated adaptive fusion mechanism, and a cross-stage gradient-injection strategy progressively propagates gradient-branch features through different levels of the encoder, enabling hierarchical perception of multiscale gradient information. Through this complementary fusion of a global semantic stream and a local gradient stream, HSCN effectively compensates for the limited sensitivity of a pure Transformer encoder to local high-frequency features.

**Figure 1:**
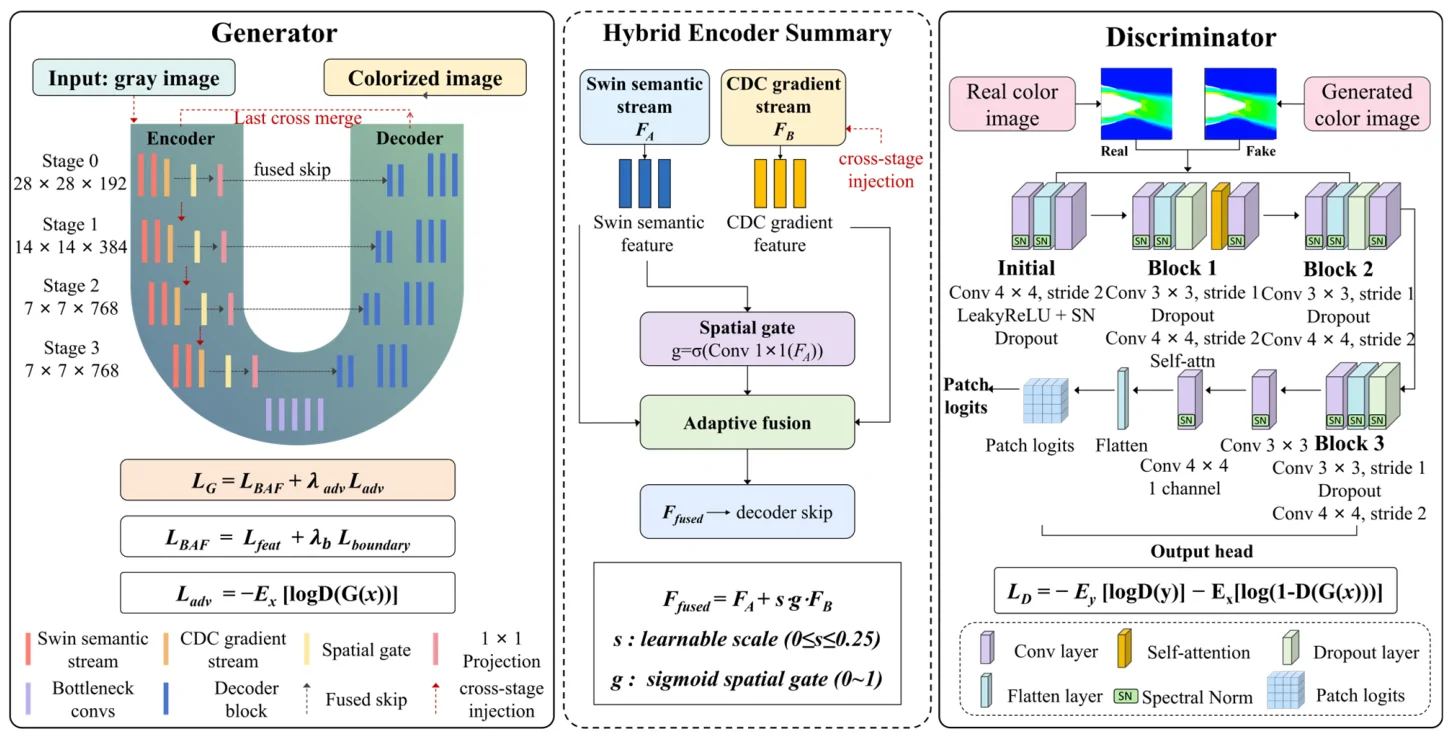
Architecture of the HSCN Model.

The discriminator adopts a multiscale deep convolutional architecture. Stacked convolutional blocks progressively reduce the feature-map resolution and expand the receptive field, allowing the authenticity of generated images to be comprehensively evaluated from local textures to the global spatial layout. Spectral normalization is applied to all convolutional layers to constrain the spectral norms of the weight matrices, limit the Lipschitz constant of the discriminator, suppress abrupt variations in discriminator outputs and gradients, improve adversarial training stability, and provide the generator with stable and reliable adversarial gradient feedback. The entire HSCN model is optimized using a two-stage strategy that combines pretraining and adversarial fine-tuning. The generator loss comprises a boundary-aware reconstruction loss and an adversarial training loss. During pretraining, the boundary-aware reconstruction loss, which combines Visual Geometry Group (VGG) perceptual features with a boundary-weighted reconstruction constraint, is the primary optimization objective, enabling the generator to recover high-frequency texture details and preserve the overall semantic structure of the image. During adversarial fine-tuning, an adversarial loss with a small weight is introduced to improve the realism of the color distribution while maintaining structural and boundary stability. This continuing competition and mutual improvement between the generator and discriminator ultimately produces colorization results with substantially better color continuity and boundary-structure fidelity, effectively suppressing color overflow across regions. The overall HSCN architecture is shown in Figure 1.

### 3.2. Hybrid Dual-Branch Encoder Module

**Figure 2:**
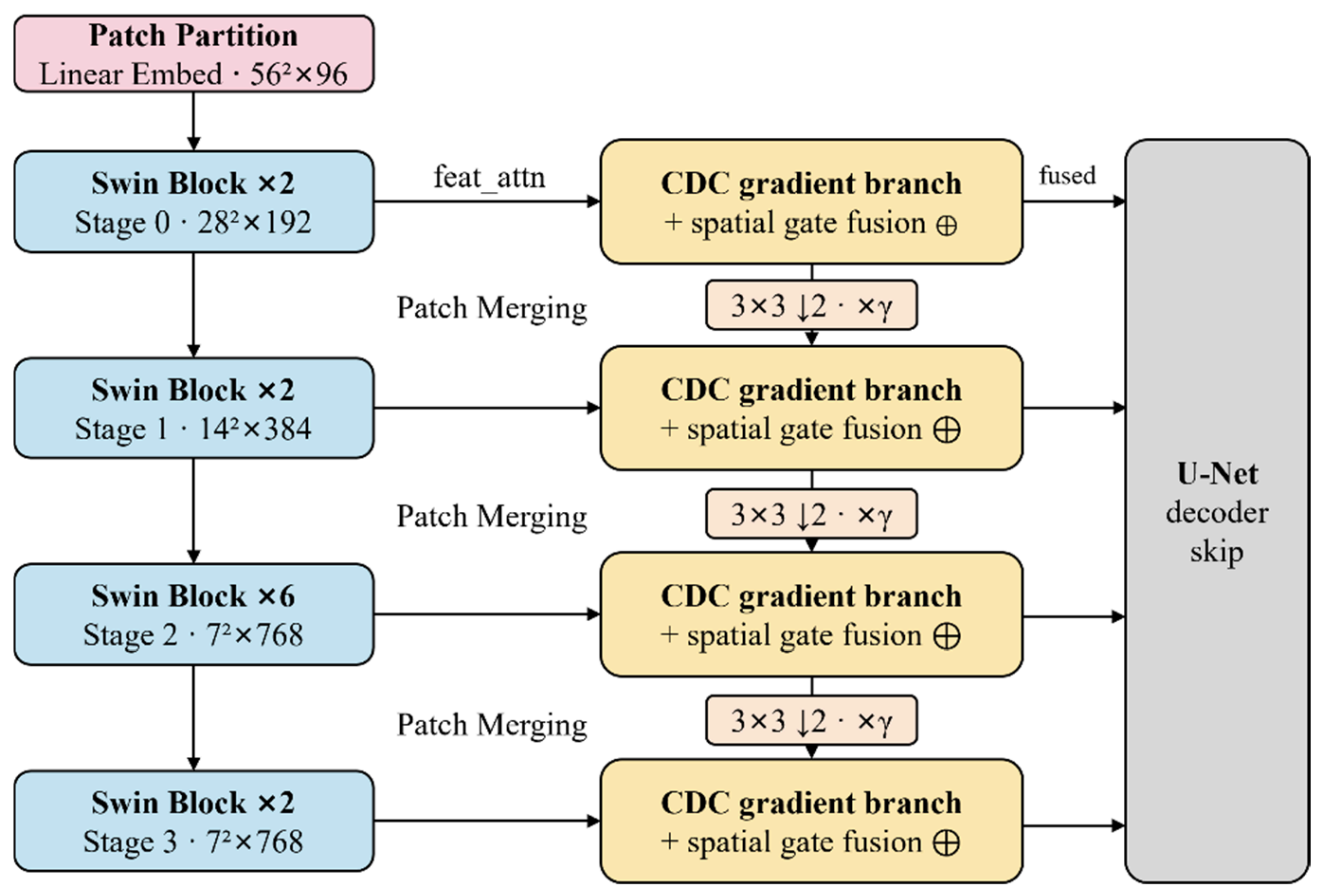
Architecture of the Hybrid Dual-Branch Encoder.

The hybrid dual-branch encoder module is the central innovation of HSCN. Existing colorization networks with CNN encoders are limited by the inherently local receptive fields of convolutional architectures and have difficulty modeling long-range temperature-distribution dependencies across flow-field image regions. Color overflow therefore readily occurs in gradient-transition regions between high- and low-temperature areas. To address this issue, this paper designs a hybrid dual-stream encoder in which a Swin Transformer semantic stream and a CDC gradient branch operate in parallel. The two streams extract global semantic information and local high-frequency gradient information, respectively, while cross-stage gradient injection and spatially gated adaptive fusion enable hierarchical feature propagation and dynamic integration. The overall module architecture is shown in Figure 2.

**Figure 3:**
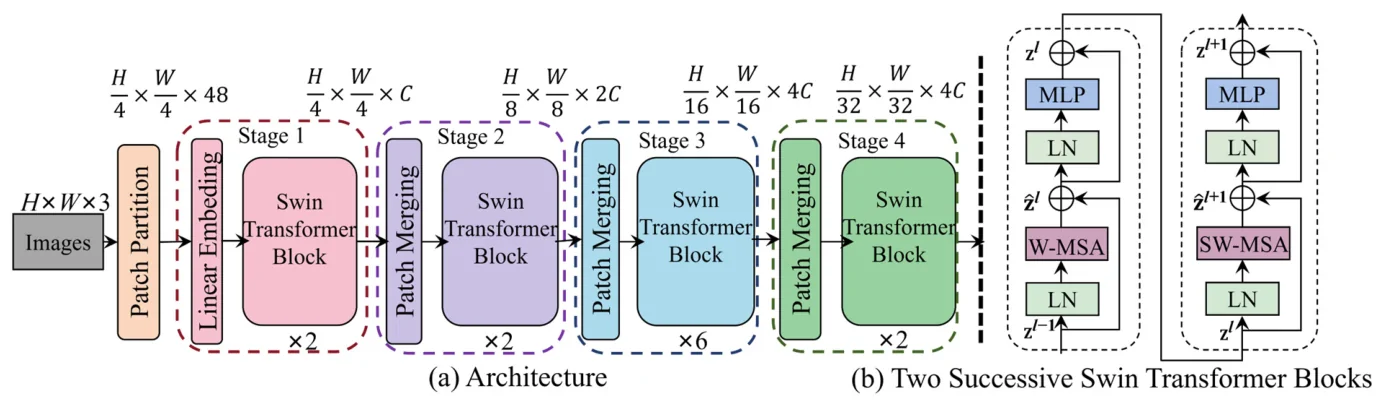
Architecture of the Swin Transformer.

The semantic stream uses the Swin Transformer as its backbone. Unlike ViT, which flattens an image into a one-dimensional sequence, the Swin Transformer adopts a hierarchical architecture and progressively downsamples feature maps through Patch Merging. Across four stages, the feature-map resolution decreases from H/8 × W/8 to H/32 × W/32, while the number of channels increases from 2C to 8C. The resulting multiscale features have different spatial resolutions and meet the skip-connection requirements of each level of the U-Net decoder. For attention computation, the Swin Transformer partitions a feature map into non-overlapping local windows, performs self-attention within each window, and enables information exchange between adjacent windows through a shifted-window mechanism. The attention is calculated as follows:

(1) $$\mathrm{Attention}(Q,K,V)=\mathrm{Softmax}(\frac{QK^{T}}{\sqrt{d}}+B)V$$

where *Q, K*, and *V* denote the query, key, and value matrices, respectively; *d* denotes the feature dimension; and *B* is a learnable relative-position encoding bias. Compared with global self-attention, window-based attention reduces the asymptotic complexity from quadratic to linear in the number of image tokens for a fixed window size, while the shifted-window mechanism enables contextual information exchange across adjacent windows. In this study, the input images were resized to 224 × 224 before being processed by the Swin Transformer. The Swin Transformer architecture is shown in Figure 3.

Although the window-based attention mechanism of the Swin Transformer offers clear advantages in modeling global semantic dependencies, it is relatively insensitive to local pixel-level high-frequency variations. Grayscale-gradient variations at boundaries in γ-photon flow-field images are often subtle, and the Swin Transformer tends to smooth this local high-frequency information during feature extraction. Consequently, boundary color overflow occurs in the generated images.

To compensate for this limitation, a gradient branch based on CDC is introduced in parallel with the semantic stream. CDC adds a pixel-difference term to standard convolution. The two operations are compared below:

Standard convolution:

(2) $$y(p_{0})=\sum_{p_{n}\in ℜ}\omega (p_{n})\cdot x(p_{0}+p_{n})$$

CDC:

(3) $$y(p_{0})=\sum_{p_{n}\in ℜ}\omega (p_{n})\cdot (x(p_{0}+p_{n})-x(p_{0}))$$

where *p*_0_ is the current pixel position, *p_n_* is a neighboring sampling point, $\omega (p_{n})$ denotes the convolution weights, and *x*(*·*) is the input feature value. The difference term makes CDC more sensitive to relative local grayscale variations than standard convolution. Because the boundaries of γ-photon flow-field images appear as slowly varying probability-density gradients after OSEM reconstruction, this gradient-sensitive formulation is used to enhance the representation of subtle boundary structures. The gradient branch consists of two sequential 3 × 3 CDC convolutions. The first is followed by batch normalization (BatchNorm) and rectified linear unit (ReLU) activation, and the second is followed by BatchNorm. The weight of the second BatchNorm layer is initialized to 0.3 to limit interference from the gradient branch with semantic-stream features at the beginning of training.

**Figure 4:**
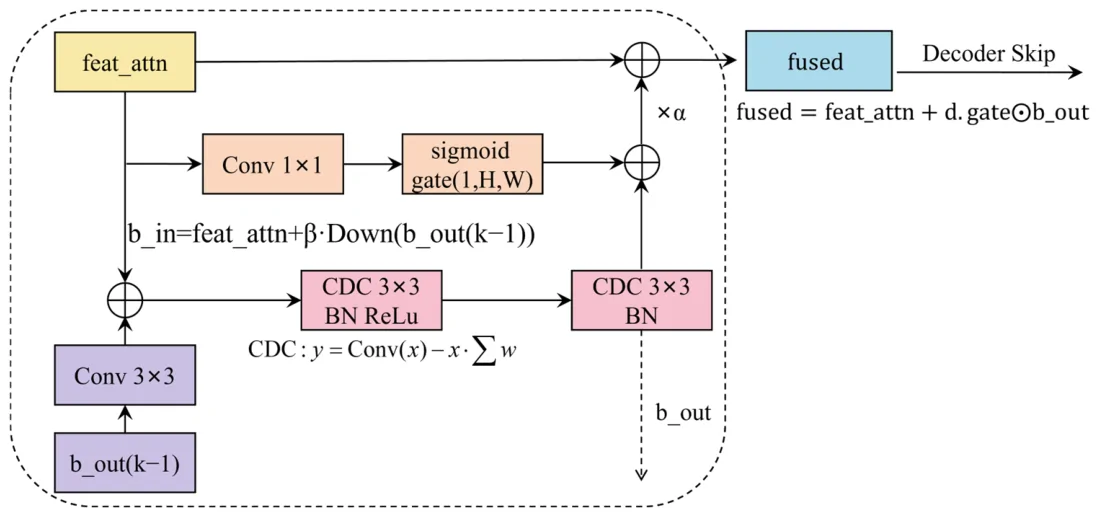
Architecture of Spatially Gated Fusion.

Because the perceptual range of the gradient branch within a single stage is limited, boundary-gradient information extracted at shallow levels cannot effectively influence deep semantic-feature representations. A cross-stage gradient-injection strategy is therefore introduced. The output of the gradient branch in stage k is downsampled using a 3 × 3 convolution with a stride of 2 and injected into the input of the gradient branch in stage k + 1, enabling gradient information to propagate progressively across encoder levels. The calculation is given by:

(4) $$B_{k+1}^{input}=B_{k+1}+\gamma_{k}\cdot {\mathrm{Conv}}_{3\times 3}^{s=2}(B_{k}^{output})$$

where *γ_k_* is a learnable injection-scaling factor with an upper bound of 0.5, which prevents an excessively strong injected signal from interfering with independent feature learning in the gradient branch of each stage. When gradient information is propagated only within a single stage, boundary features extracted at shallow levels cannot influence deep semantic representations. Consequently, the deep features still lack boundary constraints during global temperature-distribution modeling, ultimately causing overflow during color mapping. Cross-stage injection progressively transfers the gradient output of the preceding stage to subsequent stages, strengthening boundary perception as encoder depth increases and ensuring that semantic features at every level retain boundary-structure information. Specifically, a cross-stage gradient-injection mechanism is introduced between adjacent stages. The output of the gradient branch in the preceding stage is downsampled by a 3 × 3 convolution with a stride of 2 and injected into the input of the gradient branch in the next stage, allowing boundary-gradient perception to propagate progressively with encoding depth. After the semantic-stream feature F_A_ and gradient-branch feature F_B_ are obtained, they are integrated through a spatially gated adaptive fusion mechanism. The fusion architecture is shown in Figure 4.

At the beginning of training, the gradient branch has not yet fully converged. Directly adding the features of the two branches would allow unconverged gradient features to interfere with the pretrained Swin semantic features and compromise training stability. A spatial gating weight g is therefore designed. It is generated by applying a 1 × 1 convolution to the semantic-stream features, followed by a sigmoid activation function, as follows:

(5) $$g=\sigma (Conv_{1\times 1}(F_{A})+b_{gate})$$

(6) $$F_{fused}=F_{A}+s\cdot g⊙F_{B}$$

where *σ* denotes the sigmoid activation function; bgate is the gating bias and is initialized to −8.0 so that the gating weight g is close to 0 at the beginning of training, ensuring that the contribution of the gradient branch is gradually released during training rather than being imposed from the outset; s is a learnable residual-scaling coefficient with an upper bound of 0.25; and ⊙ denotes element-wise multiplication. This initialization allows the semantic stream to dominate during early training and progressively introduces gradient features as the gradient branch converges.

The hybrid dual-branch encoder module provides coordinated enhancement in both global semantic modeling and local gradient perception. The semantic stream addresses the limited receptive field of conventional CNN encoders and their inability to model long-range temperature dependencies. The gradient branch specifically compensates for the Swin Transformer’s limited sensitivity to local high-frequency information. Cross-stage injection ensures that gradient-perception capability propagates with encoding depth, while spatially gated fusion keeps integration of the two branches stable and controllable. Together, these four components allow the encoder output to contain both semantic information about the global temperature distribution and structural details of flow-field boundaries, providing a feature-level foundation for suppressing color overflow and improving boundary continuity in the colorization results.

### 3.3. Generator and Discriminator

**Figure 5:**
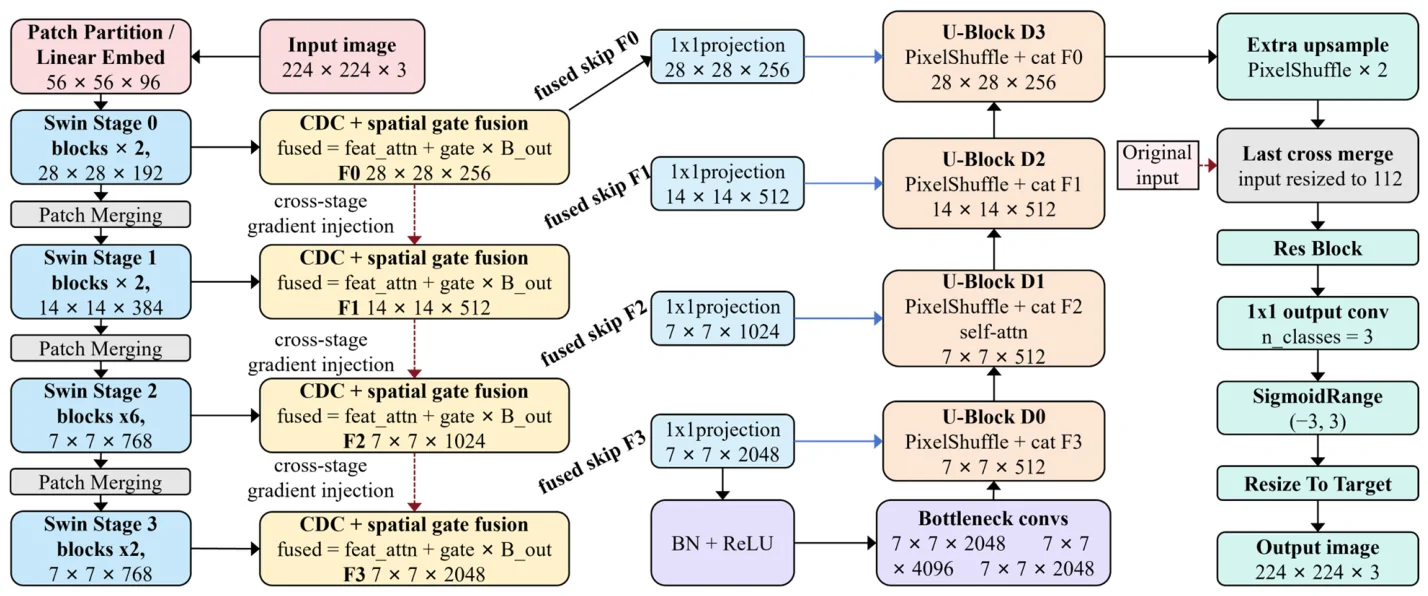
Architecture of the Generator.

The generator adopts a symmetric encoder–decoder architecture. The encoder is the hybrid dual-branch encoder module described in Section 3.2 and extracts multiscale semantic and gradient features from the original grayscale γ-photon flow-field input image. Through progressive upsampling, the decoder restores the compressed feature maps produced by the encoder to the original resolution and ultimately generates a three-channel color image. For upsampling, the initialized-to-convolution nearest-neighbor resize (ICNR) method is used to initialize the PixelShuffle weights, effectively preventing checkerboard artifacts. Two cascaded convolutional blocks are introduced at the interface between the encoder and decoder. By first expanding and then reducing the number of channels (ni → 2ni → ni), these blocks explicitly enhance cross-channel feature interactions and further refine encoder features before they enter the decoder. The generator also uses the classic U-Net skip-connection design. High-resolution features output by each encoder stage are concatenated and fused with features at the corresponding decoder level, ensuring full integration of shallow texture details and deep semantic information during decoding and preventing the loss of key structural features during repeated downsampling [24]. Finally, a 1 × 1 convolution maps the feature maps to a three-channel output, SigmoidRange (−3, 3) constrains the output to a reasonable normalized range, and ResizeToTarget restores the target size. The overall generator architecture is shown in Figure 5.

**Figure 6:**
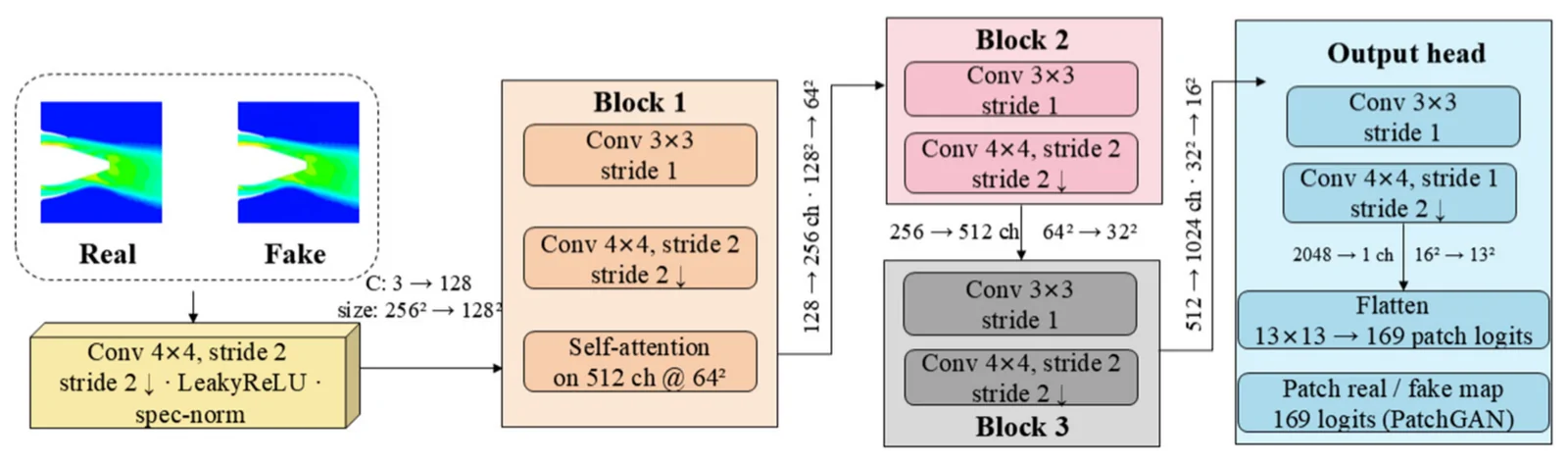
Architecture of the Discriminator.

The discriminator adopts a spectrally normalized convolutional architecture comprising multiple stacked convolutional downsampling modules and distinguishes real color images from generated color images. The discriminator receives only a three-channel real or generated color image, whereas the grayscale image is used only as the input to the generator. Each convolutional module first extracts local textures, edge details, and color-distribution features through a convolutional layer, and then downsamples the features using a convolution with a stride of 2. This process progressively reduces the spatial resolution of the feature maps while expanding the receptive field, enabling the discriminator to consider both local details and overall color consistency. To improve adversarial training stability, spectral normalization is applied to the convolutional layers to constrain the spectral norms of their weight matrices and prevent excessively large discriminator gradients from causing training oscillation. Dropout is also added to intermediate feature layers to reduce the risk of discriminator overfitting. In addition, self-attention is introduced during shallow feature extraction, enabling the model to establish dependencies between distant image regions and improving its assessment of overall structural consistency and the realism of the global color distribution. The discriminator ultimately outputs a patch-level real-or-fake response map, and the binary cross-entropy loss BCEWithLogitsLoss is used to calculate the discrimination loss. During training, an adaptive adversarial switching strategy dynamically adjusts generator and discriminator updates according to the state of the discriminator, allowing it to provide the generator with stable and effective adversarial feedback. The discriminator architecture is shown in Figure 6.

### 3.4. Loss Functions

The proposed model is optimized using a reconstruction objective during generator pretraining and an adversarial objective during fine-tuning. The boundary-aware feature loss used for pretraining consists of a perceptual feature loss and a boundary-weighted reconstruction loss:

(7) $$L_{BAF}=L_{feat}+\lambda_{b}L_{boundary}$$

where $L_{feat}$ is the multilevel perceptual feature loss, $L_{boundary}$ is the boundary-weighted reconstruction loss, and $\lambda_{b}$ is the boundary-loss weight. The perceptual feature loss not only calculates the difference between the generated and real images in pixel space but also constrains their consistency in deep feature space. It is calculated as follows:

(8) $$L_{feat}=L_{1}(\hat{y},y)+{\sum_{l=1}^{L}\lambda_{l}\left\|\phi_{l}(\hat{y})-\phi_{l}(y)\right\|}_{1}$$

where $\hat{y}=G(x)$ denotes the color image output by the generator, y denotes the real color image, $\phi_{l}(\cdot )$ denotes the output of the lth feature layer, and $\lambda_{l}$ denotes the loss weights of the different feature layers. The perceptual feature loss was computed using the ReLU3_3, ReLU4_3, and ReLU5_3 feature maps of the pretrained VGG16-BN network, with the corresponding feature-layer weights set to [20, 70, 10]. Shallow features contain local details such as edges and textures and help preserve the clarity of image boundaries and local transition regions. Intermediate features provide stronger representations of target-region structure, flow-field morphology, and the distribution of color regions and are therefore assigned a higher weight to enhance consistency in the main structure and color distribution of the generated result. Deep features primarily reflect global semantics and high-level abstractions. In this study, they mainly constrain the overall structure and are therefore assigned a relatively small weight to prevent an excessively strong high-level semantic constraint from weakening local color details. This weighting configuration provides balanced constraints across local texture, regional structure, and overall semantics, enabling the generated image to retain the flow-field structure while exhibiting a more natural color representation.

The boundary-weighted reconstruction loss uses the *L1* loss to directly measure the absolute difference between the generated and real images in pixel space:

(9) $$L_{boundary}=\frac{1}{N}\sum_{i=1}^{N}\left(1+\alpha E_{i}(y))|{\hat{y}}_{i}-y_{i}\right|$$

where $E_{i}(y)$ denotes the edge-response value of the ground-truth image at pixel *i*, α is the edge-enhancement coefficient, and *N* is the total number of pixels. The edge-response map $E_{i}(y)$ is normalized to the range of [0, 1] independently for each image. In all experiments, $\lambda_{b}$ and α were set to 1.0 and 2.0, respectively. Accordingly, the effective pixel-wise weight $1+\alpha E_{i}(y)$ adaptively varies from 1 to 3 according to the local edge strength. The setting $\lambda_{b}$ = 1.0 retains the original scale of the boundary reconstruction term, while α = 2.0 strengthens the contribution of high-response boundary pixels without eliminating the reconstruction constraint in non-boundary regions. This spatially adaptive weighting assigns greater importance to edges, contours, and regions with abrupt texture changes, thereby alleviating color overflow and boundary blurring during image colorization.

During adversarial fine-tuning, a binary cross-entropy-based adversarial loss is used. The discriminator receives real and generated color images and outputs the corresponding real-or-fake response maps. The discriminator loss is defined as:

(10) $$L_{D}=-E_{y}[\log D(y)]-E_{x}[\log(1-D(G(x)))]$$

where *D*(*y*) denotes the discriminator output for a real color image and *D*(*G*(*x*)) denotes the discriminator output for a generated color image. The generator adversarial loss is defined as:

(11) $$L_{adv}=-E\left[\log D(G(x))\right]$$

The total generator loss is:

(12) $$L_{G}=L_{BAF}+\lambda_{adv}L_{adv}$$

where $\lambda_{adv}$ is the adversarial-loss weight. During training, the boundary-aware feature loss is used as the principal optimization objective, while an adversarial loss term with a small weight is introduced to further improve the realism of the color distribution while preserving structural and boundary stability. The adversarial-loss weight is set to 0.01. Because natural colors must be restored while accurately preserving the γ-photon flow-field structure and boundary morphology, generator training remains primarily constrained by the boundary-aware feature loss, while the adversarial loss serves an auxiliary role by enhancing color realism and the naturalness of local textures. If the adversarial-loss weight is too large, discriminator feedback excessively affects the generator’s optimization direction and can cause local color shifts, boundary artifacts, or training oscillation. If the weight is too small, adversarial training cannot sufficiently improve visual realism. Setting $\lambda_{adv}$ to 0.01 allows the adversarial term to participate in generator optimization without compromising the original structural reconstruction constraint, thereby achieving a favorable balance among structural preservation, boundary clarity, and color naturalness.

## 4. Experimental Validation

### 4.1. Experimental Platform

#### 4.1.1. Data Simulation Platform

**Figure 7:**
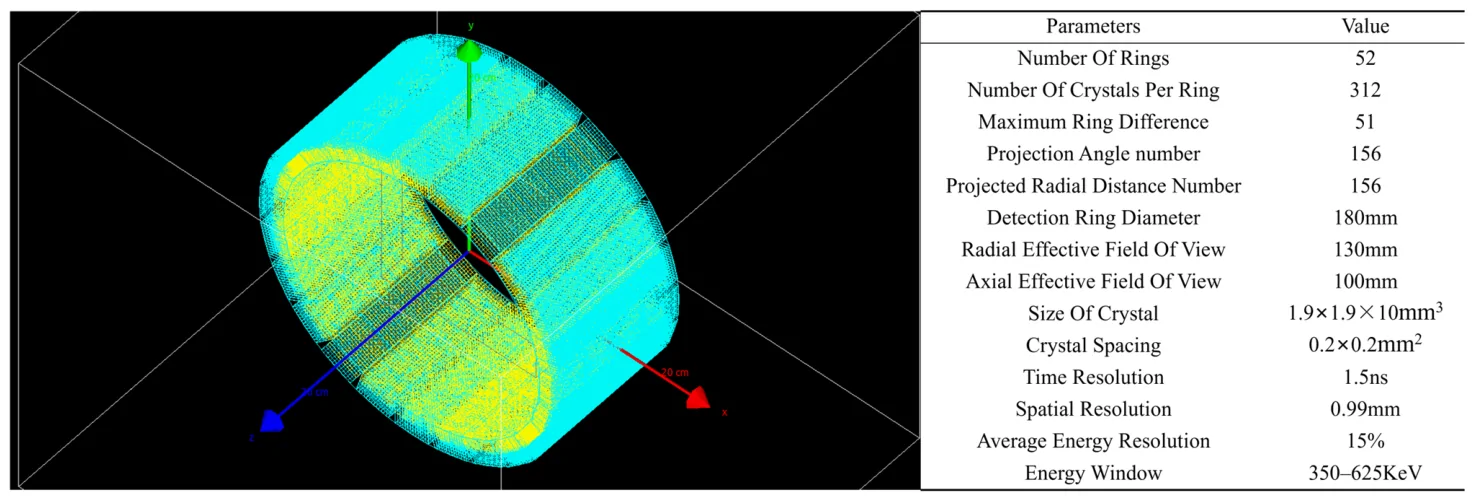
GATE-Based γ-photon Detection Simulation System and Parameters.

The flow-field temperature-distribution data used in this study were generated through ANSYS Fluent (version 2021 R1) simulations and covered several representative flow patterns, including straight jets and large-scale vortical wakes from aero-engines. These temperature-distribution fields served as ground-truth references for the subsequent colorization task. Simulations were performed using the Geant4 Application for Tomographic Emission (GATE) platform. Through Monte Carlo particle-transport modeling, GATE was used to simulate the generation, transport, and detection of γ photons. Combined with the ANSYS Fluent flow-field simulations, this framework provides a controllable and physically consistent environment for the theoretical validation of HSCN before laboratory-scale experimental testing. Flow-field temperature maps were converted into γ-photon source-density volumes, and flow-field simulations were completed by setting parameters such as photon-source activity, attenuation coefficient, time window, energy window, and acquisition time. The detector-system geometry and key physical parameters used in the simulations are shown in Figure 7. After coincidence events were acquired, the original projection data were reconstructed using the OSEM iterative algorithm, ultimately producing grayscale γ-photon flow-field images with a resolution of 128 × 128 pixels. Because OSEM reconstructions usually have limited resolution and substantial statistical noise, the convolution- and Swin Transformer-based noise-suppression super-resolution enhancement model proposed by Hui et al. was used to preprocess these grayscale γ-photon flow-field images [25]. The preprocessing model consists of a convolution-based noise-suppression module and a Swin Transformer-based super-resolution enhancement module. The OSEM-reconstructed images were first subjected to noise suppression and then enhanced to a resolution of 512 × 512. The pretrained weights obtained in the previous study were directly used in the present preprocessing stage, and the preprocessing model was not retrained or fine-tuned using the dataset of the present HSCN study. The enhanced grayscale images were then used as the preprocessed inputs for the subsequent colorization experiments.

#### 4.1.2. Data Processing Platform

The experiments were performed on a cloud graphics processing unit (GPU) server to ensure stable and reproducible training and inference. The principal computing unit was an NVIDIA A800 GPU, which provided sufficient memory to train HSCN on images with a resolution of 224 × 224, particularly given the additional computational overhead associated with the parallel Swin Transformer encoder and CDC gradient branch.

The software environment was built with Python 3.8. PyTorch (version 2.4.1+cu118) was selected as the core deep-learning framework, and the high-level FastAI application programming interface (API) was used to manage the training process, data loading, and callback mechanisms. The Swin Transformer backbone was loaded and initialized with pretrained weights using the timm library. Pillow and OpenCV were used for image reading and preprocessing, NumPy and SciPy were used for numerical computation, and image-quality evaluation metrics were implemented using scikit-image. The entire development environment was supported by CUDA 11.8 to ensure efficient GPU-accelerated computation.

### 4.2. Dataset

The original data were obtained from ANSYS flow-field simulation images of an aero-engine nozzle. However, computational fluid dynamics (CFD) simulation produces relatively few images, which is insufficient for directly training a deep generative model. This problem is more pronounced when a Swin Transformer encoder is used. Compared with CNNs, Transformer architectures lack the translation invariance and local inductive bias inherent in convolution and therefore require larger and more diverse training datasets. Insufficient data readily lead to overfitting and prevent the global modeling capability from being fully exploited. In addition, idealized CFD simulation images differ markedly from the intrinsic statistical randomness of real γ-photon tomography. Direct training on simulated images would therefore limit model generalization to real acquisition scenarios.

To address both problems, a multilevel data-augmentation scheme was designed according to the physical characteristics of γ-photon flow-field imaging, expanding the original images in terms of both sample size and statistical realism. Before data augmentation, the 42 CFD simulation cases were first divided into 27 training cases and 15 validation cases. The training and validation images were then augmented separately, and the original images and all augmented images generated from the same case were kept in the same set. Brightness and contrast adjustments simulated variations in coincidence count rates caused by radionuclide activity, detector efficiency, and acquisition conditions during actual measurements. Specifically, gamma correction factors of 1.5 and 0.75 and contrast factors of 1.5 and 0.7 were used. Gaussian blurring with a radius of 1.5 and sharpening reproduced the resolution degradation and high-frequency artifacts caused by limited temporal resolution, uncertainty in the line-of-response angle, and the statistical approximation inherent in OSEM reconstruction. Geometric transformations, including four-quadrant cropping, horizontal and vertical flipping, and rotations of ±5° and ±10°, represented variations in detector orientation and field-of-view position in industrial monitoring scenarios. Gaussian noise with a variance of 0.01 was also added to approximate image noise under low-count conditions. Each specified augmentation was generated once for each corresponding crop; therefore, no random application probability was used in this stage. Each operation was first applied to the RGB ground-truth image, and the corresponding grayscale input was then generated from the transformed image, ensuring alignment between each input and its ground truth. In addition to these fixed augmentation operations, random transformations were applied to the training images during model training. Random cropping was applied with a probability of 1.0 and horizontal flipping with a probability of 0.5. Rotation from −10° to 10°, zooming from 1.0 to 1.2, spatial warping from −0.25 to 0.25, brightness adjustment from 0.25 to 0.75, and contrast scaling from 0.5 to 2.0 were each applied with a probability of 0.75. The same randomly generated transformation parameters were applied to each paired grayscale input and RGB ground-truth image. These physics-informed augmentation methods increased the number and diversity of the training samples by introducing acquisition-related variations into the simulated images, thereby providing a broader training set for the Transformer encoder.

**Figure 8:**
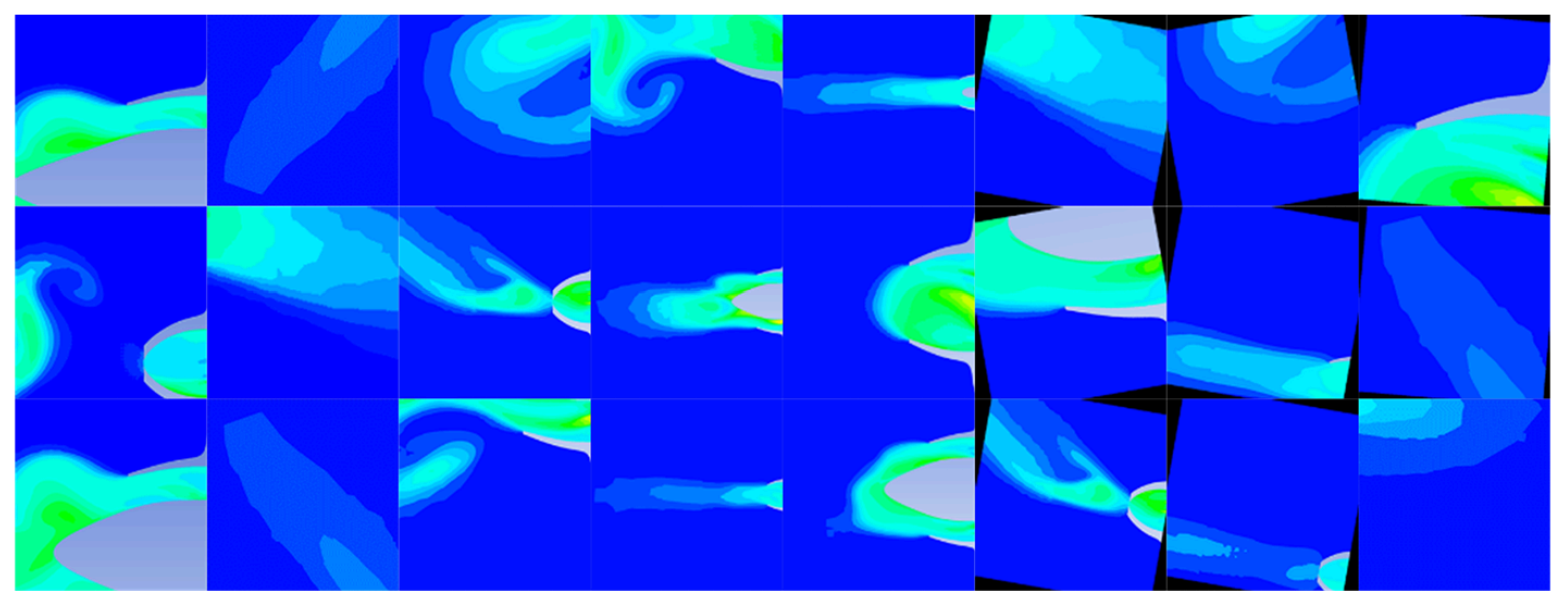
Representative RGB Ground-Truth Images from the Training Set.

**Figure 9:**
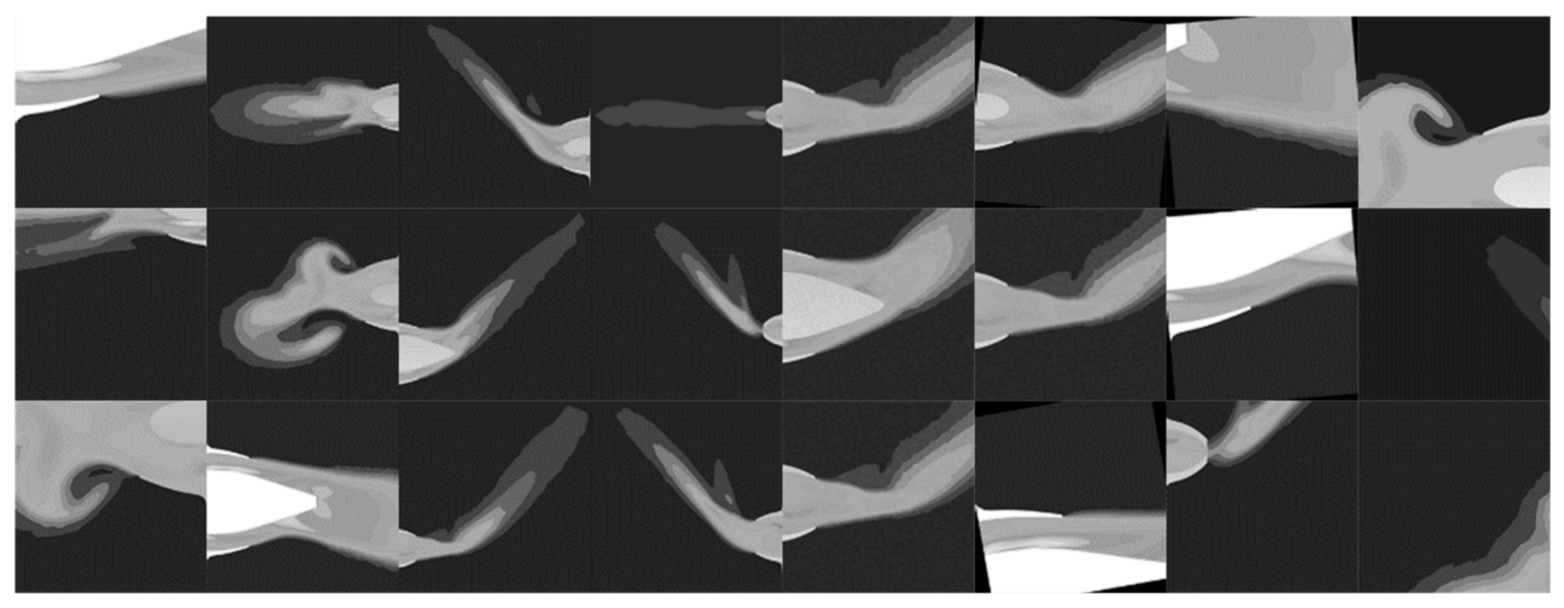
Representative Reconstructed Grayscale γ-Photon Input Images from the Validation Set.

The 42 CFD simulation cases provided 60 original input–ground-truth image pairs. Of these, 51 original pairs generated 56 augmented pairs each, seven generated 60 each, and two generated 16 each. After data augmentation, the final dataset contained 3368 images, including a training set of 2422 images and a validation set of 946 images. During training, 10% of the 2422 training images were randomly reserved as a monitoring subset using a fixed random seed, while the remaining 90% were used to update the model parameters. The monitoring subset was used to observe training loss and convergence and to select the pretraining checkpoint; it was not used to report the final performance. The validation set of 946 images was not included in this random division and was used only for the final performance evaluation after training. Large public datasets such as Common Objects in Context (COCO) and ImageNet were also introduced during training to support pretraining and further enrich the semantic priors used by the model during shallow feature extraction. Figure 8 and Figure 9 present representative RGB ground-truth images from the training set and reconstructed grayscale γ-photon input images from the validation set, respectively. Each panel represents an independent CFD simulation case, and the panels in the two figures are not arranged in one-to-one correspondence. These examples illustrate variations in flow-field orientation, wake morphology, boundary geometry, spatial position, and temperature or intensity distribution. All images were resized to 224 × 224 pixels and stored in Portable Network Graphics (PNG) format to avoid compression loss. They were normalized using the ImageNet mean and variance. The dataset used grayscale images as inputs and three-channel red-green-blue (RGB) images as outputs, consistent with the formulation of the simulated γ-photon flow-field colorization task.

### 4.3. Model Training and Results Analysis

#### 4.3.1. Model Parameter Settings

A two-stage training strategy comprising pretraining and adversarial fine-tuning was adopted.

During pretraining, images with a resolution of 224 × 224 were used as inputs; the batch size was set to 8, and a total of 300 epochs were performed. The generator used Swin Transformer Tiny (Patch4 Window7) as its encoding backbone, and an edge-gradient enhancement branch was introduced during encoding to improve the model’s perception of flow-field structural boundaries and texture details. The boundary-aware feature loss was used as the loss function. In addition to perceptual feature constraints, this loss strengthens the reconstruction constraint over boundary regions. It was intended to allow the generator to fully learn the basic grayscale-to-color mapping and establish an initial ability to perceive flow-field boundary structures before receiving adversarial signals, thereby providing a stable, high-quality model initialization for subsequent adversarial fine-tuning. Because the Swin Transformer encoder has a large number of parameters, direct end-to-end training of the complete network can lead to unstable gradients and weight oscillation at the beginning of training. A staged freezing strategy was therefore introduced during pretraining. The encoder parameters were frozen for the first 75 epochs, and only the decoder and upsampling reconstruction modules were trained, allowing the model to first learn a stable color-restoration mapping. All network parameters were then unfrozen for joint end-to-end fine-tuning, enabling further refinement of the encoder feature representations under color supervision. AdamW was used as the optimizer in conjunction with a One-Cycle learning-rate schedule to improve training stability and convergence speed. A relatively high learning rate was used during the frozen stage to accelerate decoder convergence, with the maximum learning rate set to 1 × 10^−3^. During full-model fine-tuning, the learning rate was controlled within the range of 1 × 10^−5^ to 1 × 10^−4^ to avoid substantial disruption of the pretrained backbone features. After pretraining, the checkpoint selected according to the performance on the monitoring subset was used to initialize the generator for adversarial fine-tuning.

Adversarial fine-tuning was conducted for three cycles of five epochs each, totaling 15 epochs, with a batch size of 4. The discriminator was first pretrained for two epochs at a learning rate of 5 × 10^−5^ so that it possessed basic real-or-fake discrimination capability before adversarial training began. This prevented blackened generated images or color collapse at the beginning of adversarial training due to an overly weak discriminator. The generator and discriminator were subsequently optimized alternately using an adaptive switching strategy. When discriminator accuracy exceeded the specified threshold of 0.65, the process dynamically switched to generator updates. Compared with a fixed update ratio, this adaptive mechanism better accommodates dynamic changes in discriminator capability during training and effectively maintains a dynamic balance between the two networks. The generator learning rate during the adversarial stage was further reduced to 3 × 10^−6^ to protect the Swin Transformer encoder features that had fully converged during pretraining. After the three adversarial fine-tuning cycles were completed, the generator obtained at the end of the third cycle was retained as the final HSCN model and used for all subsequent qualitative and quantitative evaluations. For reproducibility, the random seed was fixed at 42 for data partitioning, model initialization, and training, as reported in Table 1.

#### 4.3.2. Evaluation Metrics

**Figure 10:**
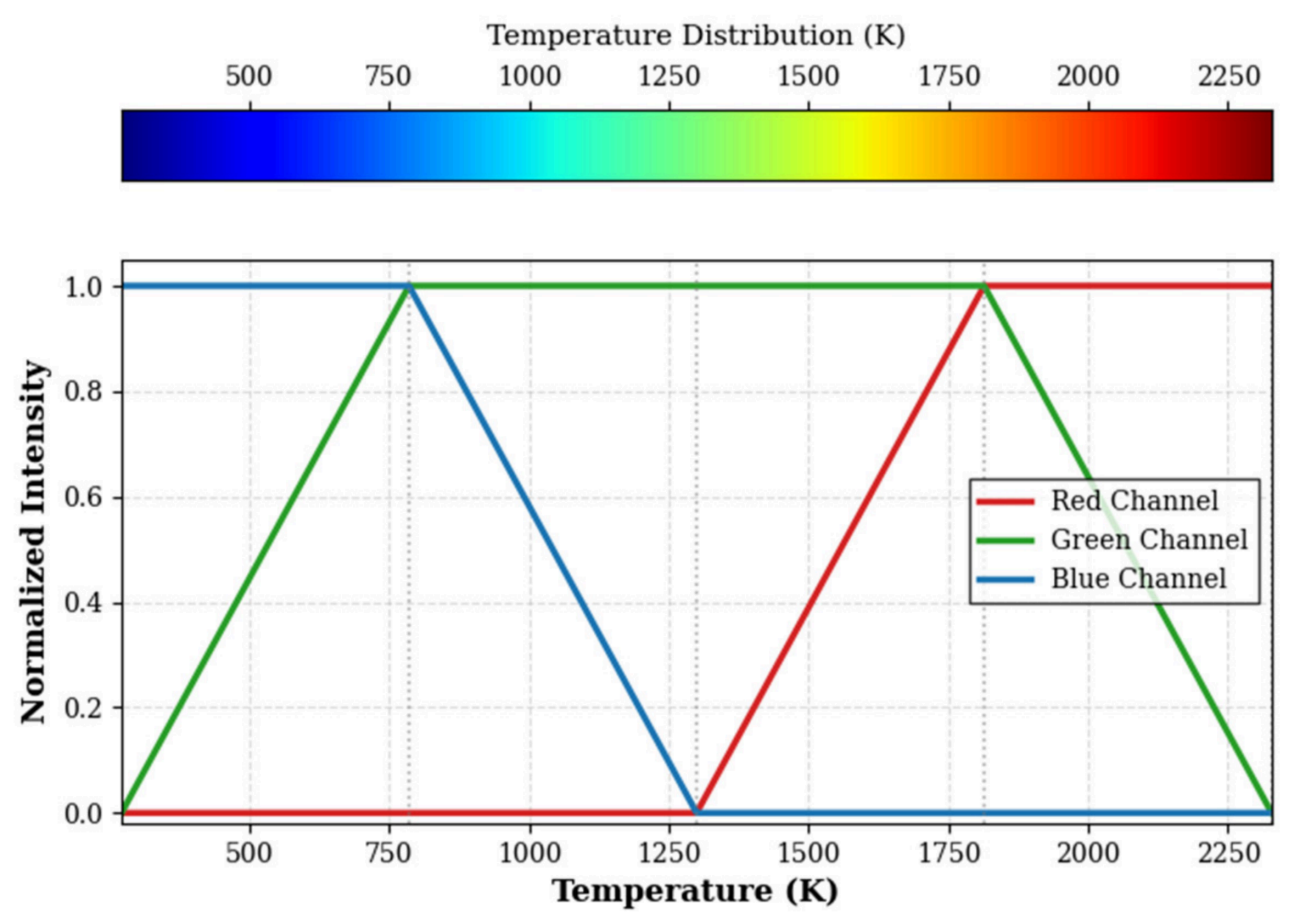
Mapping Relationship Between RGB Values and Temperature.

To comprehensively evaluate the final performance of the HSCN model, a deterministic mapping between the RGB color space and flow-field temperature in kelvin was established using the standard thermodynamic colormap from the ANSYS CFD simulation platform. As shown in Figure 10, this relationship is a piecewise linear function defined by five key anchor points: 271.3 K, 785.5 K, 1299.7 K, 1813.8 K, and 2328.0 K.

The normalized intensities of the red (R), green (G), and blue (B) channels increase and decrease linearly and alternately dominate over different temperature intervals. For example, between 785.5 K and 1299.7 K, the blue-channel intensity decreases linearly while the green-channel intensity increases linearly. This crossover between channels corresponds to complex mixed-transition regions in the flow field. The mapping model can be used to inversely map a generated pseudocolor image to a temperature field. For a localized assessment of boundary color overflow, APE was calculated between the inverted temperature and the CFD ground truth at boundary and temperature-transition locations, as follows:

(13) $$\mathrm{APE}=\left|\frac{T_{\mathrm{Pred}}(i)-T_{\mathrm{GT}}(i)}{T_{\mathrm{GT}}(i)}\right|\times 100\%$$

where $T_{GT}(i)$ denotes the CFD ground-truth temperature at the *i*-th pixel (in K), and $T_{\mathrm{Pr}ed}(i)$ is the predicted temperature inversely derived from the colorized image using the established RGB–temperature mapping function. To summarize the temperature-inversion error across the selected locations, MAPE is computed as shown in Equation (14):

(14) $$\mathrm{MAPE}=\frac{1}{N}\sum_{i=1}^{N}\mathrm{APE}$$

where N is the number of selected sampling locations. In this study, MAPE is used as a localized diagnostic for boundary-risk regions rather than as an aggregate measure over the complete evaluation set.

Peak Signal-to-Noise Ratio (PSNR) serves as a critical metric for assessing image reconstruction quality, calculated as shown in Formula (15):

(15) $$\mathrm{PSNR}=20\times \log_{10}\left(\frac{{\mathrm{MAX}}_{I}}{\sqrt{\mathrm{MSE}}}\right)$$

where MSE denotes the Mean Squared Error, calculated as shown in Formula (16):

(16) $$\mathrm{MSE}=\frac{1}{M\times N}\times \sum_{i=1}^{M}\sum_{j=1}^{N}{\left(I_{i,j}-K_{i,j}\right)}^{2}$$

where *M* and *N* denote the number of rows and columns in the image, respectively. $I_{i,j}$ represents the pixel value of the original image,$K_{i,j}$ denotes the pixel value of the reconstructed image, and ${\mathrm{MAX}}_{I}$ is the maximum value of the image pixels (typically 1.0). A higher PSNR value indicates greater similarity between the reconstructed image and the original image, signifying superior image quality.

The Structural Similarity (SSIM) Index measures the SSIM between images, as shown in Formula (17):

(17) $$\mathrm{SSIM}=\frac{\left(2\mu_{x}\mu_{y}+C_{1})(2\sigma_{xy}+C_{2}\right)}{\left(\mu_{x}^{2}+\mu_{y}^{2}+C_{1})(\sigma_{x}^{2}+\sigma_{y}^{2}+C_{2}\right)}$$

where $\mu_{x}$ and $\mu_{y}$ are the respective means of the two images, $\sigma_{x}^{2}$ and $\sigma_{y}^{2}$ are the respective variances, $\sigma_{xy}$ is the covariance, and *C*_1_, *C*_2_ are constant terms. SSIM values range between [−1, 1], with values closer to 1 indicating higher SSIM.

Mean Absolute Error (MAE) measures pixel-level error, as shown in Formula (18):

(18) $$\mathrm{MAE}=\frac{1}{N}\times \sum_{i=1}^{N}|y_{i}-{\hat{y}}_{i}|$$

where *N* is the total number of pixels, $y_{i}$ is the true pixel value, and ${\hat{y}}_{i}$ is the predicted pixel value. A smaller MAE value indicates higher pixel-level accuracy.

The Fréchet Inception Distance (FID) measures the realism and quality of generated images, as shown in Formula (19):

(19) $$\mathrm{FID}=||\mu_{r}-\mu_{g}|{|}^{2}+\mathrm{Tr}(\Sigma_{r}+\Sigma_{g}-2{\left(\Sigma_{r}\Sigma_{g}\right)}^{1/2}),$$

where $\mu_{r}$ and $\mu_{g}$ represent the mean feature values of the real and generated images, respectively, and $\Sigma_{r}$ and $\Sigma_{g}$ denote the corresponding covariance matrices. A lower FID indicates that the generated-image distribution is closer to the real-image distribution.

To determine whether the observed differences in PSNR, SSIM and MAE were consistent across independent CFD cases, the validation data were organized into 15 case-level clusters. PSNR, SSIM and MAE were first calculated for each HSCN and DeOldify output against the same ground-truth image. Before metric calculation, the outputs were resized to the same spatial resolution as the corresponding ground truth using bilinear interpolation. The metric values were then averaged within each case, giving equal statistical weight to all 15 cases. The paired case-level differences were evaluated using a two-sided exact paired sign-flip permutation test based on all 2^15^ possible sign assignments. Bias-corrected and accelerated (BCa) 95% confidence intervals were estimated using 10,000 paired case-level bootstrap resamples. For the main comparison, the *p*-values for PSNR, SSIM, and MAE were adjusted using the Holm procedure. The same case-level procedure was applied to the representative No-Fusion and No-CDC ablations. For the ablation analysis, the *p*-values from the six comparisons (two ablations × three metrics) were jointly adjusted using the Holm procedure.

### 4.4. Comparison of Experimental Results

To comprehensively evaluate the effectiveness of the proposed method, comparative experiments were conducted against mainstream methods and previous work. The comparison methods included the classic DeOldify method, the recent DDColor dual-decoder colorization network [26], and the Structure Enhancement Colorization Network (SECN) model proposed in the authors’ previous work [27]. DeOldify and DDColor represent the performance of a traditional method and a state-of-the-art technique, respectively, for general image colorization. SECN was previously proposed by our research group specifically for γ-photon flow-field image colorization and primarily addresses color-banding artifacts. It introduces a structure-enhanced attention module into the generator to alleviate staircase artifacts caused by color quantization. However, its encoder still uses a ResNet101 backbone with a limited receptive field and has difficulty modeling long-range temperature dependencies across regions. Color overflow therefore remains to varying degrees in boundary-transition regions. Comparing HSCN with these three methods verifies its advantages over general colorization algorithms on the γ-photon flow-field image task and further demonstrates the targeted improvement in boundary color overflow achieved by introducing the Swin Transformer semantic stream and CDC gradient branch into SECN.

#### 4.4.1. Analysis of the Model Training Process

**Figure 11:**
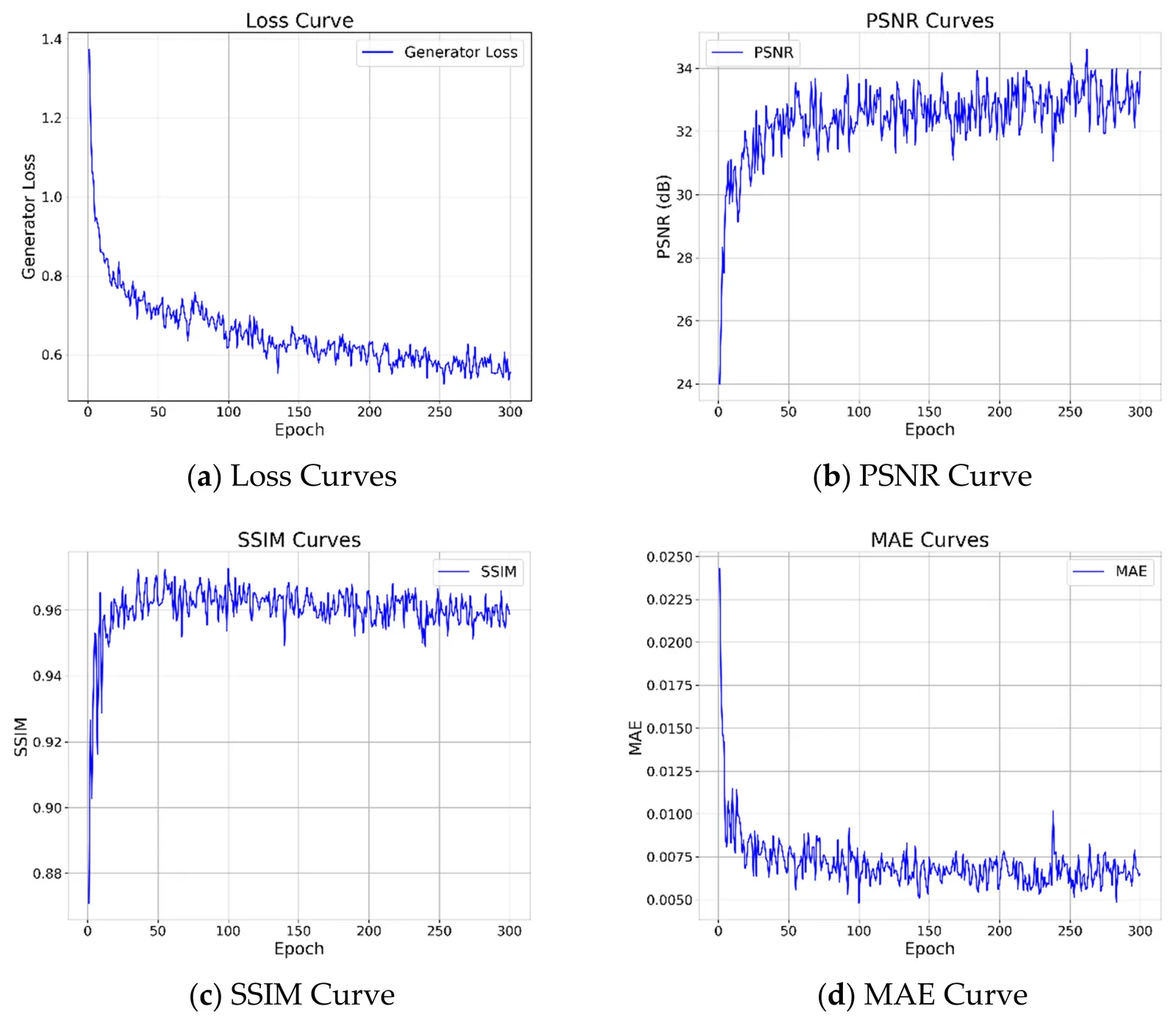
Curves for Analysis of the Generator Pretraining Process.

To evaluate convergence during generator pretraining, the generator loss calculated on the training images and the PSNR, SSIM, and MAE values calculated on the monitoring subset were recorded as functions of the training epoch, as shown in Figure 11.

Based on the metrics calculated on the monitoring subset, epoch 260 was selected from the stable late-training region, where PSNR remained near its highest level, SSIM had stabilized, and MAE remained near its minimum. The three metric curves had already entered relatively stable ranges before epoch 260, indicating that this checkpoint was not selected on the 0basis of an isolated transient fluctuation. Therefore, the checkpoint obtained at epoch 260 was selected to initialize the generator for adversarial fine-tuning. The selected generator was subsequently trained for three adversarial cycles, totaling 15 epochs, according to the strategy described in Section 4.3.1. After adversarial fine-tuning, the generator obtained at the end of the third cycle was retained as the final HSCN model. The validation set of 946 images was not involved in checkpoint selection or adversarial training and was used only for the final performance evaluation.

#### 4.4.2. Qualitative Analysis

**Figure 12:**
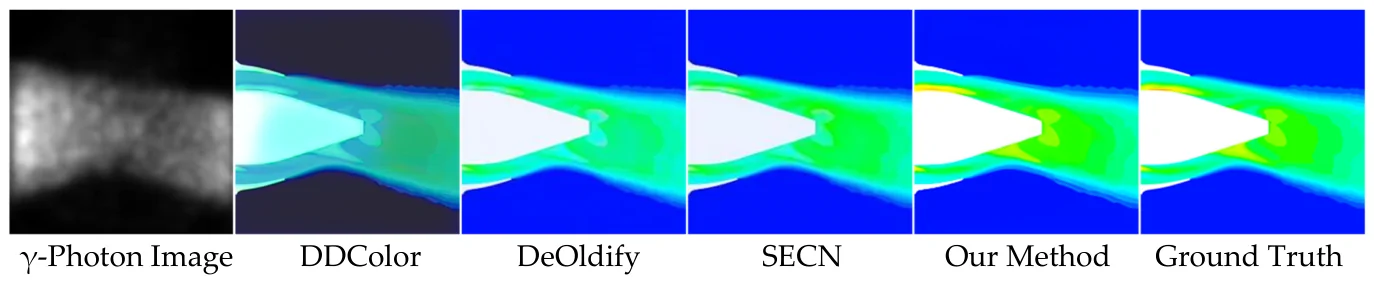
Experimental Results for Straight-Jet Wake Images.

**Figure 13:**
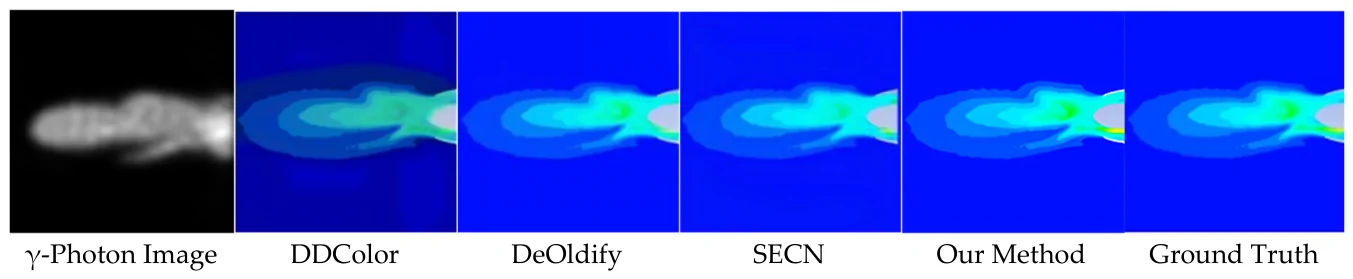
Colorization Results for the Horizontal Wake Images.

The colorization results of the different methods under the straight-jet wake and horizontal-wake conditions are shown in Figure 12 and Figure 13. Images generated by DDColor exhibit distinct color discontinuities in temperature-gradient transition zones, with unnatural color jumps and non-smooth transitions between the high- and intermediate-temperature regions. Color overflow also occurs at the interface between the main jet and the background, where structural boundaries that should be clear are incorrectly colorized, resulting in blurred contours. DeOldify exhibits similar problems, including abrupt color variations at jet boundaries, discontinuous cross-regional color transitions, and color crossing the boundary to varying degrees at the distal end of the jet. By introducing the structure-aware enhancement (SAE) module, SECN alleviates these problems. Its color transitions are smoother than those of DDColor and DeOldify, and color-banding artifacts are suppressed to some extent. However, because its encoder still uses ResNet101 and is limited by the local receptive field of convolution, slight color overflow remains at the distal end of the jet and in boundary-transition zones, and some detailed boundaries are insufficiently clear.

With the real image as the reference, the proposed HSCN method exhibits better continuity in its treatment of color gradients and higher reconstruction accuracy in preserving structural boundaries. The color transition from the high-temperature core to the edge is relatively smooth, and the levels of color attenuation at the distal end of the jet are clearly differentiated. The residual overflow observed with SECN is essentially eliminated; the interface between the main jet and the background is sharper, and the structural contour is broadly consistent with that of the real image. This improvement is mainly attributable to the Swin Transformer encoder’s ability to model long-range dependencies and thus more effectively capture temperature correlations across regions, together with the precise perception of high-frequency flow-field boundary details provided by the CDC gradient branch, which compensates for the Transformer’s limited local-information perception. Their combination improves both color continuity and boundary-structure reconstruction in HSCN.

#### 4.4.3. Comparison of Quantitative Results

After training was completed, the final model was used to perform inference on the validation set of 946 images, and the mean value of each evaluation metric was calculated. To ensure a fair comparison, all comparison algorithms were trained and tested on the same dataset using the same preprocessing procedure. The quantitative results are shown in Table 2. HSCN achieves the best result for all four metrics, namely FID, PSNR, SSIM, and MAE. DDColor performs markedly worse than the other methods for every metric because it was originally designed for natural images, whose training distribution differs substantially from that of γ-photon flow-field images, making direct adaptation to the present task difficult. DeOldify was optimized for grayscale image colorization and performs better overall than DDColor, but its ResNet101-based convolutional encoder remains inadequate for modeling long-range temperature dependencies in the flow field. SECN has already achieved good structural continuity and suppression of color banding, but HSCN further improves every metric. This finding indicates that replacing the encoder with a Swin Transformer and introducing a CDC gradient branch strengthen long-range semantic modeling and boundary-detail perception, bringing generated images closer to real images in terms of both pixel-level error and overall distributional similarity. Because flow-field temperature inversion imposes stringent requirements on color accuracy, the subsequent temperature-inversion analysis further examines whether these improvements translate into lower inversion errors under the same simulation-based setting.

A case-level paired statistical analysis was further conducted across the 15 independent CFD cases. HSCN achieved a case-level mean PSNR of 36.5597 ± 2.0813 dB, compared with 32.7068 ± 1.6531 dB for DeOldify, with a mean paired improvement of 3.8530 dB (95% BCa CI: 3.5963–4.3705). For SSIM, HSCN and DeOldify achieved case-level mean values of 0.9073 ± 0.0296 and 0.8538 ± 0.0297, respectively, corresponding to a mean paired improvement of 0.0535 (95% BCa CI: 0.0487–0.0570). For MAE, HSCN achieved 0.01310 ± 0.00360, compared with 0.01830 ± 0.00384 for DeOldify, corresponding to a mean paired reduction of 0.00520 (95% BCa CI: 0.00469–0.00574). HSCN outperformed DeOldify in all 15 cases for all three metrics. All three confidence intervals excluded zero, and all differences remained significant after Holm correction across the three metrics (adjusted *p* = 0.000183). These results indicate systematic and consistent differences across the evaluated cases rather than case-to-case sampling variation. The case-level macro averages differ from the image-level averages in Table 2 because each CFD case was assigned equal statistical weight in the paired analysis.

While the preceding case-level paired analysis evaluates the consistency of HSCN’s performance across independent CFD cases for the reference seed−42 run, it does not assess the run-to-run variability introduced by model initialization and stochastic training. Therefore, two additional independent training runs of the complete HSCN model were conducted using random seeds of 45 and 48. The dataset partition, monitoring subset, network configuration, training schedule, and evaluation set were kept identical across all three runs. As summarized in Table 3, the seed−42, seed−45, and seed−48 runs yielded consistently comparable performance. Across the three runs, the mean ± sample standard deviation was 10.7542 ± 0.0193 for FID, 38.2809 ± 0.5209 dB for PSNR, 0.9332 ± 0.0046 for SSIM, and 0.0102 ± 0.0016 for MAE. All three runs retained the same overall performance pattern, and HSCN outperformed DeOldify on all four metrics in each run. These results indicate that the observed performance advantage was not specific to the original seed−42 initialization and provide direct empirical evidence of repeatability across the three evaluated random seeds.

Values in the final row are reported as the mean ± sample standard deviation across the three independent training runs.

#### 4.4.4. Comparison of Temperature Inversion Results

**Figure 14:**
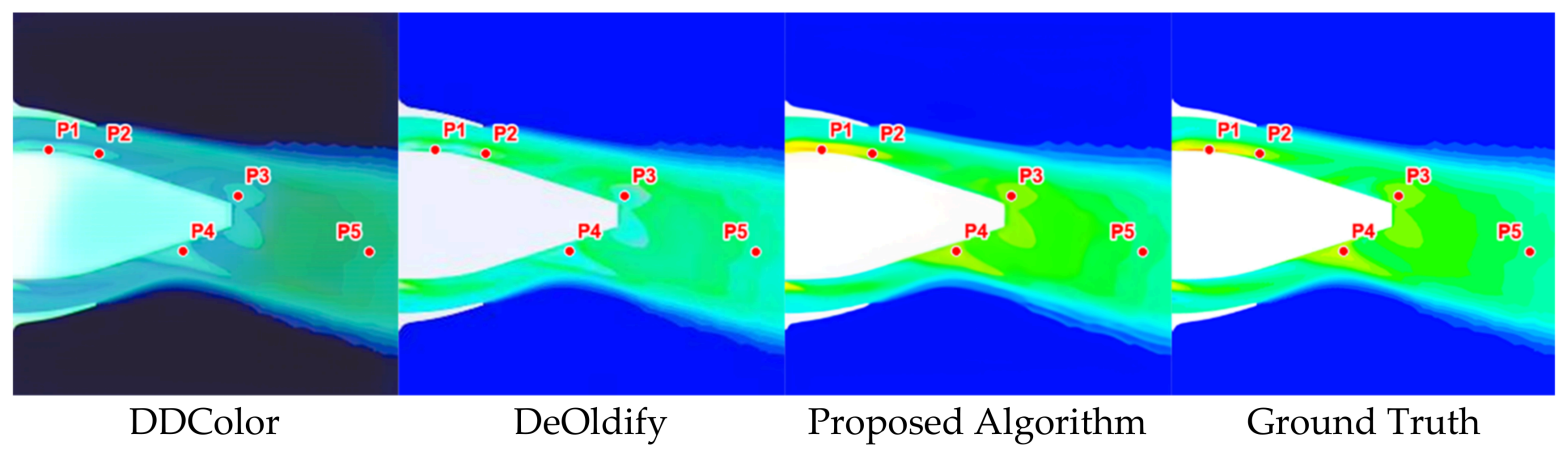
Example Temperature Inversion Sampling points.

To verify the color-restoration accuracy of each method, a temperature-inversion experiment was conducted using fixed-point sampling on three challenging samples selected from the evaluation set. These samples cover different flow-field structures, and their image-level PSNR and SSIM values are reported in Table 4. For each sample, five sampling points, P1 to P5, were selected in key regions, including the high-temperature core, temperature-transition regions, and jet boundaries. Figure 14 shows the sampling locations in Sample 1. The sampling locations in Samples 2 and 3 were selected according to the same regional criteria. To reduce sensitivity to individual-pixel fluctuations and interpolation noise, the RGB value at each location was obtained as the channel-wise median within a fixed 3 × 3 neighborhood. For each sample, identical sampling coordinates were used for the ground-truth image and all colorization results. The established RGB-to-temperature mapping was then used to convert the sampled RGB values into temperature readings. For each sample, MAPE was calculated over five sampling locations, and the Total Average MAPE was calculated over all 15 sampling locations. The results are presented in Table 4.

The data in Table 4 show that HSCN consistently achieves the lowest MAPE across all three samples. Specifically, the MAPE values obtained by HSCN are 2.74%, 12.00%, and 23.20% for Samples 1, 2, and 3, respectively, and are lower than those obtained by DeOldify and DDColor in each case. Although the three samples exhibit different image-level PSNR and SSIM values, HSCN maintains relatively consistent advantages in temperature inversion. When all 15 sampling points are considered, HSCN achieves a Total Average MAPE of 12.65%, compared with 31.24% for DeOldify and 28.70% for DDColor, corresponding to reductions of 18.59 and 16.05 percentage points, respectively. Overall, Table 4 provides a localized descriptive assessment of temperature-inversion performance at the evaluated boundary and temperature-transition locations. Because the five locations within each sample are nested measurements rather than independent experimental units, the point-level MAPE values are reported descriptively rather than treated as independent observations for statistical significance testing.

In Sample 1, P4 is located at the interface between the main jet and the background, where boundary color overflow is most likely to occur. At this point, neither DeOldify nor DDColor correctly restores the local color. Both incorrectly fill the region with a cooler tone, resulting in a substantial deviation between the inverted and ground-truth temperatures. In contrast, the HSCN inversion result at this point is close to the ground truth, indicating that it restores color and structure more accurately in boundary regions. This result corroborates the qualitative finding that HSCN produces clearer boundaries without color overflow. The experimental results demonstrate that HSCN achieves high accuracy in both structural and color restoration and can provide more reliable inputs for downstream flow-field temperature inversion.

### 4.5. Ablation Studies

To verify the effectiveness of the proposed components, ablation experiments were performed on the key components of the hybrid dual-branch encoder module, and the rationale for the design was evaluated by quantifying the contribution of each component to overall performance. The experiments included five configurations: the complete model, a model without the CDC gradient branch (No-CDC), a model without spatially gated adaptive fusion (No-Gate Fusion), a model without cross-stage gradient injection (No-Cross Stage), and a model without both gated fusion and cross-stage injection (No-Fusion). The No-CDC configuration removed the entire gradient branch, reducing the encoder to only the Swin Transformer semantic stream. The No-Gate-Fusion configuration retained the CDC gradient branch and cross-stage gradient injection but removed the spatially gated adaptive fusion mechanism. The No-Cross-Stage configuration retained the CDC gradient branch and gated fusion but removed cross-stage gradient injection. The No-Fusion configuration retained the gradient branch but removed both spatially gated fusion and cross-stage injection, causing the gradient and semantic features to be fused through direct addition.

**Figure 15:**
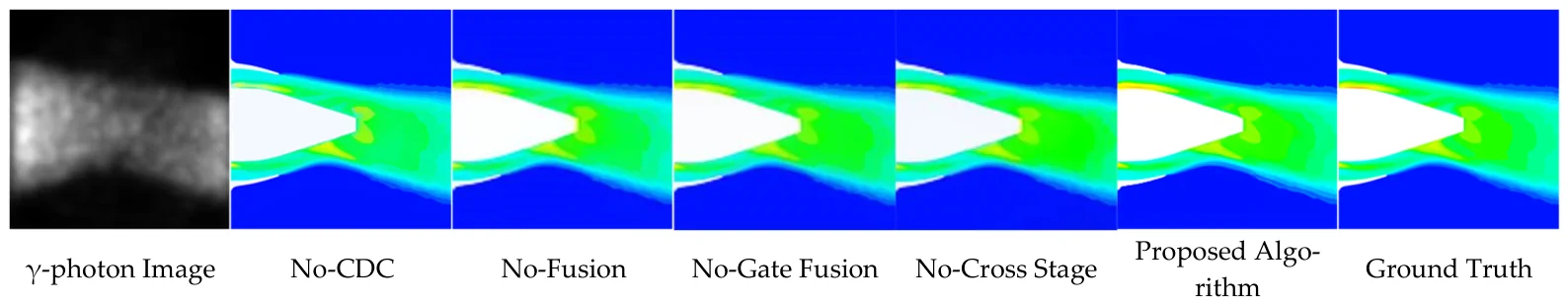
Qualitative Analysis of the Ablation Experiments.

As shown in Figure 15, the No-CDC configuration lacks the supplementary high-frequency boundary information provided by the gradient branch. It therefore exhibits relatively pronounced color overflow in temperature-transition regions and insufficiently clear structural contours, demonstrating that the CDC gradient branch plays a key role in restoring the detailed structure of flow-field boundaries. Although the No-Fusion configuration retains the gradient branch, it lacks a gating mechanism that adaptively regulates the fusion ratio. Unconverged gradient features therefore interfere with the semantic stream, producing color instability and slight structural misalignment in some regions. The No-Gate Fusion configuration also exhibits reduced boundary consistency, indicating that the spatial gating mechanism is important for adaptively regulating the contribution of gradient features during fusion. In the No-Cross Stage configuration, the boundary transition becomes less distinct, suggesting that cross-stage gradient injection helps propagate boundary-gradient information to deeper encoder levels. The more pronounced degradation observed in the No-Fusion configuration further indicates that the two mechanisms provide complementary support for stable dual-stream feature integration. The complete model performs well in both respects, producing smooth color transitions and clear boundary structures that most closely resemble the real image.

The quantitative results of the ablation experiments are shown in Table 5. The quantitative results show that removing the proposed components generally degrades reconstruction performance, as reflected by decreases in PSNR and SSIM and increases in MAE. In particular, the complete HSCN achieves the highest PSNR and SSIM and the lowest MAE, demonstrating the best overall reconstruction accuracy and structural fidelity. It should be noted that the No-CDC configuration achieves an FID of 10.6821, which is slightly lower than the 10.7344 obtained by the complete model. However, its PSNR decreases from 38.7422 to 33.8580, SSIM decreases from 0.9372 to 0.8839, and MAE increases from 0.0085 to 0.0173. This indicates that the CDC gradient branch primarily contributes to pixel-level reconstruction accuracy and structural preservation, while its effect on the distribution-level similarity measured by FID is comparatively limited. The metrics deteriorate more substantially for the No-Fusion configuration, further confirming that the introduction of a gradient branch must be accompanied by an appropriate fusion mechanism to fully exploit the advantages of the dual-stream architecture. Otherwise, feature conflicts can instead impair model performance.

To further assess whether the performance changes produced by representative key ablations were consistent across cases, additional paired analyses were conducted for the No-Fusion and No-CDC configurations. Compared with No-Fusion, the complete HSCN improved PSNR by 4.0664 dB (95% BCa CI: 3.8257–4.6332) and SSIM by 0.0604 (95% BCa CI: 0.0540–0.0636), while reducing MAE by 0.00435 (95% BCa CI: 0.00384–0.00472). Compared with No-CDC, HSCN improved PSNR by 1.4656 dB (95% BCa CI: 1.2194–2.0780) and SSIM by 0.0117 (95% BCa CI: 0.0086–0.0146), while reducing MAE by 0.00237 (95% BCa CI: 0.00197–0.00280). HSCN achieved better results in all 15 cases for all six comparisons, and all differences remained significant after Holm correction across the six tests (adjusted *p* = 0.000366). Under the reported fixed-seed setting, these results provide case-level statistical support for the contributions of the fusion design and the CDC gradient branch.

**Figure 16:**
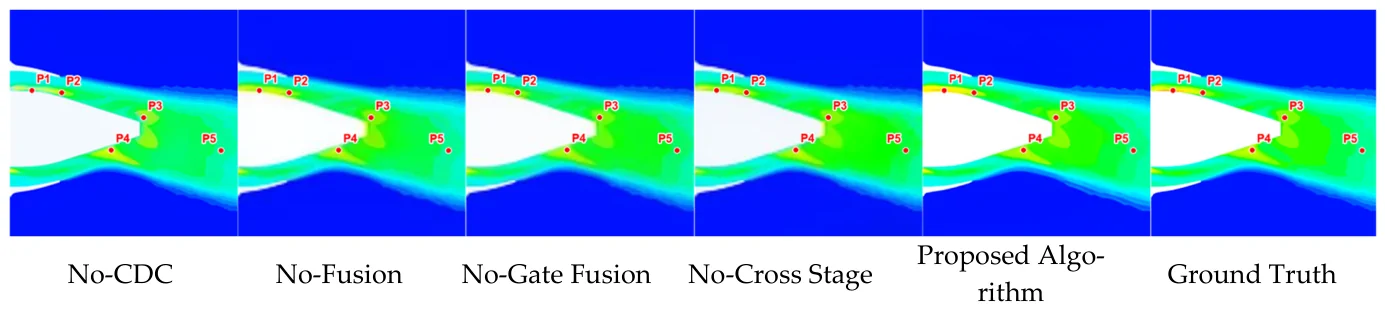
Sampling Points for Temperature Inversion in the Ablation Study.

Temperature-inversion analysis was performed on the ablated models. The sampling points used for physical-experiment inversion are shown in Figure 16.

The quantitative temperature-inversion results of the ablation experiments are shown in Table 6. The No-CDC, No-Gate Fusion, No-Cross Stage, No-Fusion, and complete-model configurations were evaluated at five sampling points, P1 to P5, in each of the three samples. The MAPE was calculated separately for each sample, and the Total Average MAPE was calculated over all 15 sampling points.

The results in Table 6 show that the complete model achieves the lowest MAPE for all three samples, with values of 2.74%, 12.00%, and 23.20%, respectively, and the lowest Total Average MAPE of 12.65%. The corresponding Total Average MAPE values of No-CDC, No-Gate Fusion, No-Cross Stage, and No-Fusion are 22.74%, 15.38%, 20.95%, and 16.28%, which are 10.09, 2.73, 8.30, and 3.63 percentage points higher than that of the complete model, respectively. Among the ablated configurations, No-CDC yields the highest Total Average MAPE, indicating that the CDC gradient branch plays an important role in temperature recovery at flow-field boundaries and temperature-transition regions. Removing cross-stage injection increases the Total Average MAPE to 20.95%, supporting the contribution of progressive gradient-information propagation across encoder stages. The higher Total Average MAPE values of No-Gate Fusion and No-Fusion further demonstrate the contribution of spatially gated feature integration. Overall, these results support the complementary contributions of the CDC gradient branch, cross-stage injection, and spatially gated fusion to temperature-inversion accuracy at the evaluated boundary and temperature-transition locations.

## 5. Conclusions

To address the boundary color overflow commonly observed in grayscale γ-photon flow-field image colorization, this paper proposes a γ-photon flow-field image colorization algorithm based on the HSCN. The algorithm is built on a conditional generative adversarial network framework. In the generator, a hybrid dual-stream encoder comprising a Swin Transformer semantic stream and a CDC gradient branch replaces the conventional ResNet101 backbone. The discriminator adopts a multiscale convolutional architecture constrained by spectral normalization. The generator and discriminator work jointly to enhance structural integrity and suppress high-frequency artifacts caused by noise from probabilistic statistical imaging. Within the hybrid encoder, a cross-stage gradient-injection strategy progressively propagates gradient information through the semantic stream and gradient branch, while a spatially gated adaptive fusion mechanism dynamically integrates features from the two branches. The model thereby retains global semantic modeling capability while remaining sensitive to high-frequency flow-field boundary structures, effectively alleviating color diffusion across regions. Simulation-based experimental results demonstrate that the algorithm performs well in evaluations of γ-photon flow-field colorization, achieving PSNR, SSIM, FID, and MAE values of 38.7422, 0.9372, 10.7344, and 0.0085, respectively. Compared with DeOldify, PSNR and SSIM are improved by 24.30% and 11.89%, while FID and MAE are reduced by 42.98% and 60.47%, respectively. Furthermore, in the reference seed−42 run, case-level paired analysis across 15 validation cases showed statistically significant improvements in PSNR and SSIM and a statistically significant reduction in MAE over DeOldify, with all three 95% confidence intervals excluding zero and all Holm-adjusted *p*-values below 0.001. The ablation experiments, together with the case-level paired analyses of the representative No-Fusion and No-CDC configurations, provide statistical support for the contributions of the CDC gradient branch and the overall fusion design, while the component-specific effects of spatially gated fusion and cross-stage injection are reflected in the corresponding ablation results in Table 4 and Table 5. In the temperature-inversion analysis, HSCN achieves a Total Average MAPE of 12.65% across 15 boundary and temperature-transition locations in three representative samples, compared with 31.24% for DeOldify and 28.70% for DDColor. These results further demonstrate that HSCN improves temperature-recovery accuracy at boundary and temperature-transition locations. The conclusions of this study therefore concern the experimentally evaluated improvements in color-restoration accuracy, boundary reconstruction, and temperature-inversion performance; no comparative claim is made regarding computational efficiency or deployment cost. A systematic computational-cost analysis across the compared architectures will be considered in future work, using standardized profiling protocols and hardware settings. However, the present study remains at the theoretical-validation stage. The ANSYS Fluent and GATE simulation frameworks were used to verify the feasibility and effectiveness of HSCN under controlled and physically consistent simulation conditions. A laboratory-scale γ-photon imaging platform for high-temperature and high-pressure environments with synchronized detection is currently under development. Future work will first validate HSCN using data acquired from this platform and will subsequently examine its applicability to real aero-engine flow-field monitoring.

## Figures and Tables

**Table 1:** Model Training Parameters and Hyperparameter Configuration.

Parameters	Value
Network Factor	2
Optimizer	AdamW
Random Seed	42
Weight Decay	${1\times 10}^{-3};\;$ ${1\times 10}^{-2}$
Pretrain Batch Size	8
Pretrain Epochs	300
Frozen-stage Max LR	${1\times 10}^{-3}$
Fine-tuning LR Range	${1\times 10}^{-5}$~${1\times 10}^{-4}$
GAN Batch Size	4
GAN Epochs	15

**Table 2:** Quantitative Analysis of Colorization Performance.

	FID ↓	PSNR ↑	SSIM ↑	MAE ↓
DeOldify	18.8261	31.1684	0.8376	0.0215
DDColor	49.6979	14.0462	0.6219	0.1369
SECN	17.8541	32.5831	0.8612	0.0191
Proposed Algorithm	10.7344	38.7422	0.9372	0.0085

**Table 3:** Quantitative Analysis of Random-Seed Performance.

Random Seed	FID ↓	PSNR ↑	SSIM ↑	MAE ↓
42	10.7344	38.7422	0.9372	0.0085
45	10.7554	37.7159	0.9282	0.0117
48	10.7729	38.3845	0.9343	0.0104
Mean ± SD	10.7542 ± 0.0193	38.2809 ± 0.5209	0.9332 ± 0.0046	0.0102 ± 0.0016

**Table 4:** Quantitative Analysis of Temperature Inversion.

	HSCN PSNR (dB)	HSCN SSIM	DeOldify MAPE	DDColor MAPE	Proposed Algorithm MAPE
Sample 1	28.4116	0.9440	38.08%	41.82%	2.74%
Sample 2	33.4824	0.9753	25.43%	15.06%	12.00%
Sample 3	29.3711	0.9686	30.22%	29.22%	23.2%
Total Average	-	-	31.24%	28.70%	12.65%

**Table 5:** Quantitative Analysis of Ablation Experiments.

	FID ↓	PSNR ↑	SSIM ↑	MAE ↓
No-CDC	10.6821	33.8580	0.8839	0.0173
No-Fusion	36.4931	31.5139	0.8346	0.0189
No-Gate Fusion	36.8215	31.5827	0.8489	0.0183
No-Cross Stage	37.1406	31.5106	0.8404	0.0188
Proposed algorithm	10.7344	38.7422	0.9372	0.0085

**Table 6:** Quantitative Analysis of Temperature Inversion in the Ablation Study.

	No-CDC MAPE	No-Gate Fusion MAPE	No-Cross Stage MAPE	No-Fusion MAPE	Proposed Algorithm MAPE
Sample 1	16.35%	7.57%	10.30%	5.35%	2.74%
Sample 2	22.66%	13.84%	24.79%	20.22%	12.00%
Sample 3	29.22%	24.73%	27.77%	23.27%	23.2%
Total Average	22.74%	15.38%	20.95%	16.28%	12.65%

## Data Availability

The γ-photon flow-field images used in the study come from ANSYS FLUENT and GATE simulation experiments. The datasets used and/or analyzed in the current study are available from the corresponding author upon reasonable request. Our team has released the code as open source and uploaded it to a public code hosting platform. Correspondence and requests for materials could be addressed to Xiao Hui.
